# Identification and Functional Characterization of Genes Involved in the Biosynthesis of Caffeoylquinic Acids in Sunflower (*Helianthus annuus* L.)

**DOI:** 10.3389/fpls.2019.00968

**Published:** 2019-07-31

**Authors:** Ketthida Cheevarungnapakul, Gholamreza Khaksar, Pawinee Panpetch, Patwira Boonjing, Supaart Sirikantaramas

**Affiliations:** ^1^Molecular Crop Research Unit, Department of Biochemistry, Faculty of Science, Chulalongkorn University, Bangkok, Thailand; ^2^Molecular Sensory Science Center, Faculty of Science, Chulalongkorn University, Bangkok, Thailand

**Keywords:** hydroxycinnamoyl-coenzyme A:quinate hydroxycinnamoyl transferase, hydroxycinnamoyl-coenzyme A:shikinate/quinate hydroxycinnamoyl transferase, caffeoylquinic acid, sunflower (*Helianthus annuus* L.), sprout, functional characterization

## Abstract

Sunflower (*Helianthus annuus* L.) sprouts accumulate high amounts of caffeoylquinic acids (CQAs) including chlorogenic acid (5-CQA) and 1,5-diCQA. These compounds, which can be found in many plants, including tomato, globe artichoke, and chicory, have many health benefits, including antioxidant, antihepatotoxic, and antiglycative activities. However, CQA profiles and biosynthesis have not previously been studied in sunflower sprouts. In the present study, we found that 5-CQA and 1,5-diCQA were the major CQAs found in sunflower sprouts. We also identified minor accumulation of other CQAs, namely 3-CQA, 4-CQA, 3,4-diCQA, and 4,5-diCQA. According to genome-wide identification and phylogenetic analysis of genes involved in CQA biosynthesis in sunflower, three genes (*HaHQT1*, *HaHQT2*, and *HaHQT3*) encoding hydroxycinnamoyl CoA:quinate hydroxycinnamoyl transferase (HQT) and two genes (*HaHCT1* and *HaHCT2*) encoding hydroxycinnamoyl CoA:shikimate/quinate hydroxycinnamoyl transferase (HCT) were identified. Expression analysis of these five genes in hypocotyls and cotyledons strongly suggested that HaHQT2 could be the main enzyme responsible for CQA biosynthesis, as *HaHQT2* had the highest expression levels. In addition, when transiently expressed in the leaves of *Nicotiana benthamiana*, all three HaHQTs, which were soluble and not membrane-bound enzymes, could increase the content of 5-CQA by up to 94% compared to that in a control. Overall, our results increase understanding of CQA biosynthesis in sunflower sprouts and could be exploited by plant breeders to enhance accumulation of health-promoting CQAs in these plants.

## Introduction

**Figure 1:**
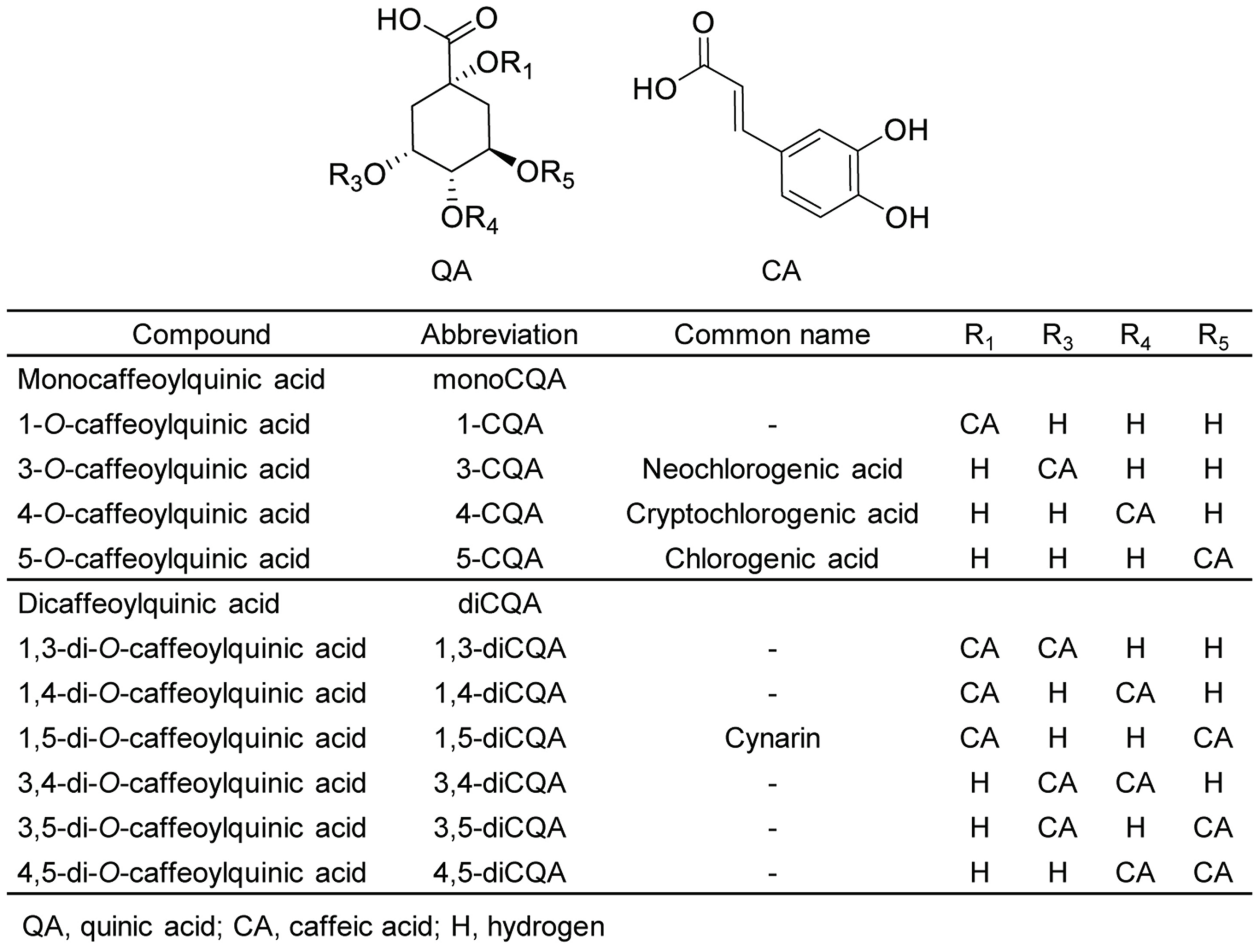
Structures of caffeoylquinic acids found in plants. The names, abbreviations, common names, and chemical structures of caffeoylquinic acid derivatives, including monocaffeoylquinic acids (monoCQAs) and dicaffeoylquinic acids (diCQAs) are shown.

Increased health consciousness among consumers and concerns about the negative health effects of chemical preservatives used in the food industry has led to increase an interest in natural and herbal substances. Fruits and vegetables accumulate a wide range of bioactive compounds with many health-promoting benefits. Among these bioactive compounds, phenolics are of high importance, and caffeoylquinic acids (CQAs) comprise one of the most common phenolic groups. When caffeoyl moieties combine with quinic acid, CQAs are formed. These CQAs can be categorized into various groups based on the position, number, and identity of their acyl group. The monocaffeoylquinic acid (monoCQA) group includes 1-CQA, 3-CQA (known as neochlorogenic acid), 4-CQA (known as cryptochlorogenic acid), and 5-CQA (known as chlorogenic acid). The dicaffeoylquinic acid (diCQA) group includes 1,3-diCQA, 1,4-diCQA, 1,5-diCQA, 3,4-diCQA, 3,5-diCQA, and 4,5-diCQA (Figure 1).

CQAs can be found in numerous plant species, including *Cichorium intybus* (chicory; Legrand et al., 2016), *Cynara cardunculus* L. var. *scolymus* (globe artichoke; Moglia et al., 2016), and *Helianthus annuus* L. (sunflower; Sun et al., 2012) in the family Asteraceae; *Solanum lycopersicum* L. (tomato) and *Nicotiana tabacum* L. (tobacco) in the family Solanaceae (Niggeweg et al., 2004); *Coffea* spp. (coffee; Lallemand et al., 2012) in the family Rubiaceae; and *Ipomoea batatas* L. (sweet potato; Kojima and Kondo, 1985) and *Ipomoea aquatica* (water spinach; Lawal et al., 2016) in the family Convolvulaceae. The antioxidant, anti-inflammatory, anti-hypertension, and antimicrobial properties of CQAs are well documented by previous studies. In addition, several *in vitro* and *in vivo* studies have shown additional benefits of CQAs, such as a reduction in the risk of cardiovascular diseases (de Sotillo and Hadley, 2002), hepatoprotective properties (Salomone et al., 2017), and inhibition of HIV replication and integration (McDougall et al., 1998; Gu et al., 2007). Notably, CQAs can confer resistance to abiotic stressors such as UV light (Cle et al., 2008) and to biotic stressors (Niggeweg et al., 2004; Leiss et al., 2009; Legrand et al., 2016) in plants. The bioactivity of CQAs mainly depends on their isomerization. For example, the number and position of caffeic acid moieties in diCQAs affect their antioxidant properties (Xu et al., 2012).

Sunflower (*Helianthus annuus* L.) seeds and sprouts are rich in phenolic compounds and vitamins and thus exhibit a wide variety of potential health-beneficial characteristics, including anti-inflammatory, antimicrobial, antioxidant, antihypertensive, and wound-healing properties (Fowler, 2006; Bashir et al., 2015; Guo et al., 2017). In a study by Weisz et al. (2009), monoCQA and diCQA contents comprised up to ~3,359 and 460 mg per 100 g dry weight of seed kernels, respectively. Among the 11 phenolic compounds analyzed in sunflower seed kernels, 5-CQA was the most abundant. In another study by Sun et al. (2012), the antiglycative and antioxidant characteristics of four edible sprouts were investigated, and it was found that sunflower sprout extract exhibited similar antiglycative properties compared with aminoguanidine, a well-known synthetic antiglycative agent. The strong antioxidant and antiglycative properties of sunflower sprouts were attributed to their rich 1,5-diCQA content. Pająk et al. (2014) examined the effect of germination on nutritional value (total phenolics and flavonoids) and antioxidant properties of sunflower seeds. Interestingly, they found that total phenolics, flavonoids, and antioxidant capabilities were significantly higher in sunflower sprouts than seeds. Moreover, HPLC profiling of sunflower phenolics revealed that CQA content was 3.7-fold higher in sprouts than seeds. Taken together, these observations prompted us to investigate sunflower sprouts for our study.

**Figure 2:**
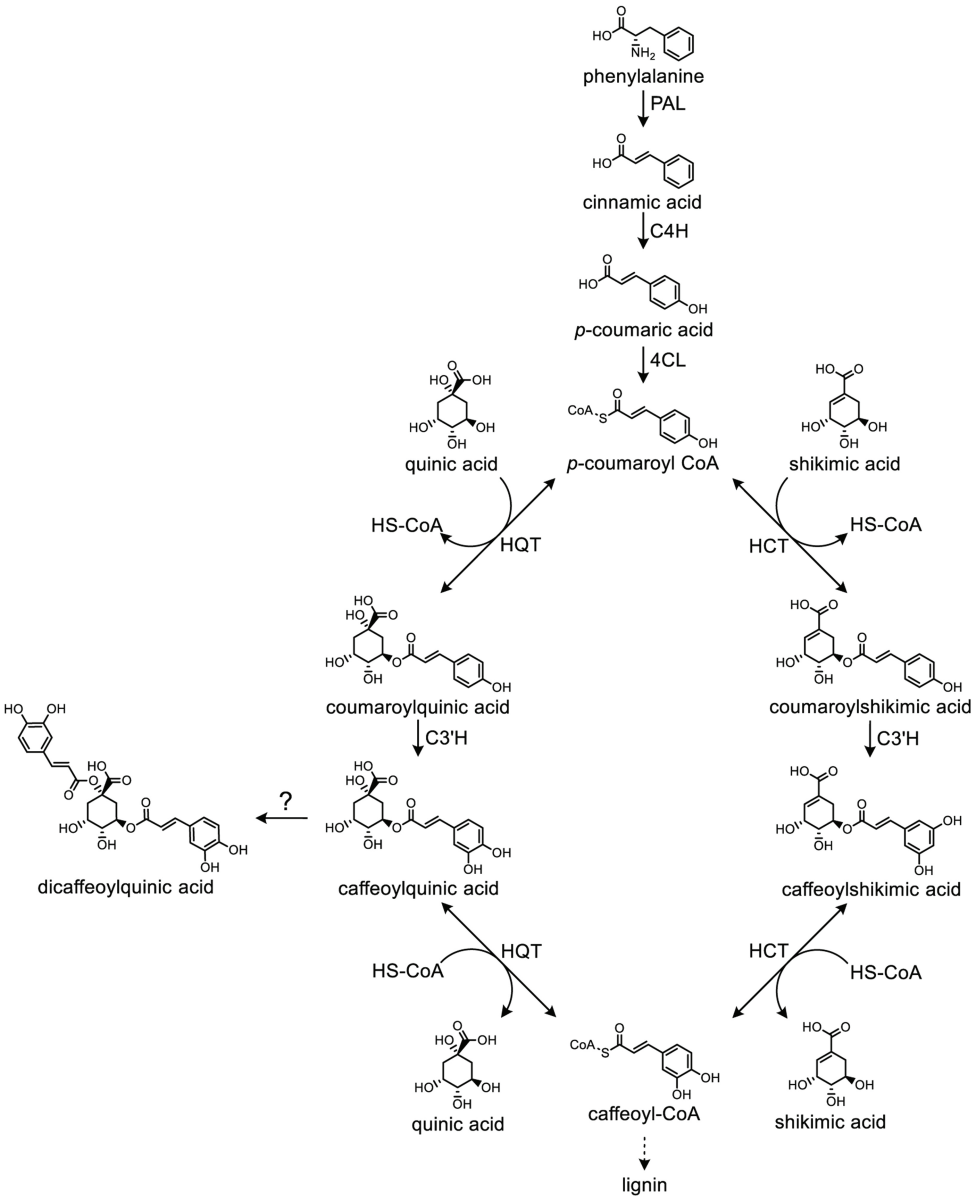
Biosynthetic pathway of caffeoylquinic acids in plants. The roles of phenylalanine ammonia lyase (PAL), cinnamate 4-hydroxylase (C4H), 4-hydroxycinnamoyl-CoA ligase (4CL), hydroxycinnamoyl-CoA:quinate hydroxycinnamoyl transferase (HQT), hydroxycinnamoyl-CoA:shikimate/quinate hydroxycinnamoyl transferase (HCT), and p-coumaroyl ester 3′-hydroxylase (C3′H) are depicted.

CQAs are biosynthesized *via* the phenylpropanoid pathway in plants (Comino et al., 2009). The starting point of this pathway is the aromatic amino acid phenylalanine (Phe), which is deaminated by phenylalanine ammonia lyase (PAL) to form cinnamic acid. Then, cinnamate-4-hydroxylase (C4H) and 4-coumarate coenzyme A ligase (4CL) sequentially convert cinnamic acid to form *p*-coumaroyl-CoA. Two possible routes have been proposed for the next step in CQA synthesis. In the first route, hydroxycinnamoyl-CoA:quinate hydroxycinnamoyl transferase (HQT) converts *p*-coumaroyl-CoA to coumaroylquinate, which is then hydroxylated by *p*-coumarate-3′-hydroxylase (C3′H) to form CQA (Niggeweg et al., 2004; Comino et al., 2009; Menin et al., 2010). In the alternative second route, hydroxycinnamoyl-CoA:shikimate/quinate hydroxycinnamoyl transferase (HCT) catalyzes the formation of *p*-coumaroylshikimate from *p*-coumaroyl-CoA. The *p*-coumaroylshikimate is subsequently hydroxylated to caffeoylshikimic acid by C3′H (Mahesh et al., 2007; Moglia et al., 2009). Notably, HQT and HCT both catalyze reversible reactions (Figure 2).

HQT and HCT enzymes belong to the BAHD superfamily of plant-specific acyl-CoA-dependent acyltransferases (Yu et al., 2009; Tuominen et al., 2011). However, our knowledge regarding the biosynthesis of diCQAs is still limited. In a study by Lallemand et al. (2012), a recombinant HCT enzyme cloned from coffee was shown to synthesize diCQAs from 5-CQA. In addition, the enzyme HQT was reported to convert 5-CQA to diCQAs in tomato (Moglia et al., 2014). Legrand et al. (2016) identified two HCTs (HCT1 and HCT2) and three HQTs (HQT1, HQT2, and HQT3) in chicory. Notably, increased levels of 3-CQA were detected in *N. benthamiana* leaves transiently expressing HQT1 or HCT1. Moreover, genes involved in CQA biosynthesis, including *HQT*, *HCT*, *C3*′*H*, *Acyltransf_1*, and *Acyltransf_2*, have been isolated and characterized in artichoke (Comino et al., 2007, 2009; Moglia et al., 2009; Menin et al., 2010). Moglia et al. (2016) found two *HCT*s and three *HQT*s in artichoke, which is the same number of *HCT*s and *HQT*s found chicory. To the best of our knowledge, there are currently no published studies in the literature on how CQAs are formed during sprouting stages and how high the CQA content is. Therefore, we focused on sunflower sprout, which is currently gaining popularity among health-conscious consumers. In addition, none of HQTs and HCTs have been identified in sunflower. Acquiring a deeper understanding of CQA biosynthetic enzymes in sunflower is critically important to aid efforts to biofortify sunflower sprouts as functional foods. In the present study, we report the identification and functional characterization of key genes involved in CQA biosynthesis in sunflower sprouts.

## Materials and Methods

### Plant Material and Growth Conditions

Sunflower (*H. annuus* L.) seeds were purchased from a local supplier (“Super Top,” Thailand). The seeds were first washed, soaked in tap water for 8 h, and wrapped with wet cheesecloth overnight. Next, they were germinated on coconut dust at 30°C under 60% relative humidity for 9 days, with dark conditions for the first 48 h, followed by a 12/12 h light/dark photoperiod for the remaining days. The seed coat was gently removed at hour 56 (after exposure to the light for 8 h; Supplementary Figure S1A). To compare metabolites and genes expression of the sample obtained from different developmental stages and tissues, the experiment was divided into two sets. For the first set, whole plants excluding roots were collected at different time points (days 3–9, 2 h after light exposure at each day; Supplementary Figure S1B) after germination. For the second set, cotyledons and stems were collected similarly at day 5. For both sets, there were five replicates per time point, and each replicate consisted of 10 sprouts. Replicates were collected separately, frozen in liquid nitrogen, ground into a fine powder using an MM400 mixer mill (Retsch®, Germany) at 30 Hz for 1 min, and then stored at −80°C until further use. In addition, half of the samples were also freeze dried for HPLC analysis.

To grow five-week-old plants for an agroinfiltration experiment, *N. benthamiana* seeds were sown on peat moss and grown in a controlled-climate room at 25°C and with a 16/8 h light/dark photoperiod (artificial light of 4,500 Lux). Two-week-old plants were transplanted individually into pots and were left to continue growing under the same conditions.

### Determination of Caffeoylquinic Acids Contents of Plant Tissues

To analyze CQA contents of plant tissues, 20 mg dry weight of sunflower sprout tissue and 20 mg fresh weight of *N. benthamiana* leaves were extracted with 1 ml of 80% (v/v) methanol containing an internal standard, 0.05 g L^−1^ puerarin. The reactions were mixed vigorously at 15°C for 15 min by shaking at 1,500 rpm and then centrifuged at 12,000 × *g* for 15 min. Supernatant was collected and filtered through 0.2 μm nylon syringe filters.

A Shimadzu UFLC system equipped with an SPD-M20A photodiode array detector (Shimadzu, Japan) and Kinetex® C18 (250 mm × 4.6 mm, 5 μm; Phenomenex®, USA) was used to analyze 10 μl of the extract from sunflower sprouts and *N. benthamiana* leaves. Chromatographic separation was performed using 0.1% (v/v) TFA in water (solvent A) and 0.1% (v/v) TFA in acetonitrile (solvent B) as the mobile phase. The following elution gradient was used: 5% B for 5 min, 5–15% B for 10 min, a 25-min hold, 15–100% B for 4 min, a 2-min hold, 100–5% B for 4 min, and a 5-min hold. The flow rate was set at 1.5 ml min^−1^, and the column oven temperature was maintained at 40°C. UV spectra were acquired in the range of 190–800 nm, and chromatograms were obtained at 320 nm. Peaks corresponding with the retention time and UV spectrum of a commercial standard were identified as CQAs. Amounts of each CQA were calculated according to the calibration curve in the range of 0.5–0.007825 mg ml^−1^. Puerarin was used as an internal standard (Sigma-Aldrich, USA). All CQA standards used in this study were purchased from Carbosynth, England.

Additionally, to confirm identities of the CQAs, the components were analyzed using an Agilent UHPLC system (Agilent Technologies, USA) using Kinetex® C18 (250 mm × 4.6 mm, 5 μm; Phenomenex®, USA). The following elution gradient was used: 0–5% B for 5 min, 5–15% B for 30 min, a 65-min hold, 15–100% B for 5 min, a 5-min hold, 100–5% B for 5 min, and a 10-min hold. The flow rate was set at 0.5 ml min^−1^, and the column oven temperature was maintained at 40°C. For MS/MS analysis, QTRAP® 4,500 MS/MS System (AB Sciex™, USA) in multiple reaction monitoring (MRM) and negative ionization mode (ESI-) was used. Operating conditions for MS analysis were as follows: heat block temperature of 500°C, curtain nitrogen gas 30 psi, nebulizer and auxiliary gases of 50 psi, collision nitrogen gas at medium position, ionization voltage of −4,500 V, and entrance potential (EP) of −10. For the tested compounds, the following transition under optimal instrumental conditions of collision energy (CE) of −35 eV, declustering potential (DP) of −50 V, and collision cell exit potential (CXP) of −12 V.

### Identification of Putative *HQT* and *HCT* Genes in Sunflower (*HaHQT*s and *HaHCT*s)

The HQT of tomato (*Solanum lycopersicum*; NP_001234850.2; Moglia et al., 2014) was used as a query for tBlastn search against the sunflower genome database HA412HO bronze assembly^1^. The open reading frames of *HQT* and *HCT* were identified. Then, using EMBL-EBI Clustal Omega (McWilliam et al., 2013), the amino acid sequences of putative HaHQTs and HaHCTs were aligned with well-characterized HQTs/HCTs belonging to different plant species (Supplementary Figure S2). These candidates including *HaHQT1* (accession number MK598073), *HaHQT2* (accession number MK598074), *HaHQT3* (accession number MK598075), *HaHCT1* (accession number MK598076), and *HaHCT2* (accession number MK598077) were selected for further study.

### Phylogenetic Analysis

Amino acid sequences of putative HaHQTs and HaHCTs were aligned with sequences of previously characterized enzymes using BioEdit ClustalW multiple alignment (Hall, 1999), and a Neighbor Joining (NJ) tree was created using MEGA7 software (Kumar et al., 2016) with 1,000 bootstrapped data sets.

### RNA Isolation, cDNA Synthesis, and Cloning of Putative *HaHQT*s

Total RNA was isolated from 100 mg fresh weight of sunflower sprouts using TRI reagent® (Molecular Research Center, Inc., USA). Next, RNA concentration and integrity were analyzed by measuring A_260_ and A_280_ on an Eppendorf Biophotometer® D30 (Eppendorf, Germany) and by agarose gel electrophoresis, respectively. The RNA was treated with RNase-free DNase I (Thermo Fisher Scientific, USA), and then, the first strand cDNA was synthesized by RevertAid Reverse Transcriptase using oligo(dT)_20_ primers (Thermo Fisher Scientific, USA) according to the manufacturer’s instructions.

The full-length putative *HaHQT*s were amplified with Phusion Hot Start II High-Fidelity DNA Polymerase (Thermo Fisher Scientific, USA) using the prepared cDNA of sunflower sprout as a template. Then, the amplified DNA was cloned into pCR™8/GW/TOPO®TA vectors (Invitrogen, USA) resulting in pCR™8/GW/TOPO®-*HaHQT*s and subsequently sequenced. One clone of each putative gene was used for further study (sections Promoter Analysis and Transient Overexpression of *HaHQT*s in *N. benthamiana*).

### Gene Expression Analysis of Sunflower Sprout

Total RNA was extracted from sunflower sprouts as described above (section RNA Isolation, cDNA Synthesis, and Cloning of Putative *HaHQT*s). Then, qRT-PCR was performed using gene-specific primers (Supplementary Table S1). Eukaryotic translation initiation factor 5A (*ETIF5A*; XM_022156448.1), elongation factor 2 (*EF2*; XM_022137686.1), and actin 7 (*ACT7*; XM_022154554.1) of sunflower were used as reference genes (Ochogavía et al., 2017). Reactions were conducted in volumes of 10 μl in a 96-well PCR plate using Luna® universal qPCR master mix (New England Biolabs®, USA). A CFX Connect™ Real-Time PCR Detection System and CFX Manager™ Software (BIO-RAD, USA) were used to conduct PCR, and melting curve analysis was used to confirm the existence of a single product. Relative expression level of each gene was calculated using 2^−ΔCt^ (Schmittgen and Livak, 2008) according to the average Ct values of three reference genes (Beekman et al., 2011).

For droplet digital PCR (ddPCR), a 20-μl reaction mixture containing gene-specific primers (Supplementary Table S1), QX200™ ddPCR™ EvaGreen Supermix (BIO-RAD, USA), and cDNA was generated as a droplet with QX200™ Droplet Generation Oil for EvaGreen (BIO-RAD, USA) using QX200™ droplet generator (BIO-RAD, USA). EF2 was used as a reference gene. The PCRs were performed in a 96-well PCR plate using a T100™ Thermal Cycler (BIO-RAD, USA). After amplification, QX200™ Droplet Reader (BIO-RAD, USA) was used to measure the fluorescence intensity of each individual droplet. Absolute transcript levels (copies/20 μl reaction) were processed using QuantaSoft™ Software (BIO-RAD, USA). Relative transcript number of each gene was presented as a ratio of the absolute transcript levels (copies/20 μl reaction) of the target gene to the reference gene (Taylor et al., 2017).

### Promoter Analysis

The 2,000 bp upstream regions of start codon of putative HaHQTs were *in silico* scanned for regulatory elements using MatInspector (Cartharius et al., 2005). The genomic localization of the analyzed promoters was Chr10: 227227684…227229684 for *HaHQT2* and Chr2: 166074069…166076069 for *HaHQT3*.

### Transient Overexpression of *HaHQT*s in *Nicotiana benthamiana*

The putative *HaHQT*s from pCR™8/GW/TOPO®-*HaHQT*s were transferred into pEAQ-HT-DEST1 (pEAQ1) expression vectors (Peyret and Lomonossoff, 2013) using Gateway® LR Clonase® II (Invitrogen, USA). The resultant pEAQ1-*HaHQT*s were then transformed into *Agrobacterium tumefaciens* LBA4404 by electroporation.

*A. tumefaciens* colonies containing each construct were grown in 25 ml of LB broth containing 50 mg L^−1^ kanamycin, 50 mg L^−1^ streptomycin, and 50 mg L^−1^ rifampicin and shaken at 250 rpm at a temperature of 30°C overnight. Cells were harvested by centrifugation at 3,000 × *g* for 10 min and washed in MM buffer twice (10 mM MES and 10 mM MgCl_2_, pH 5.6). Then, the pellet was resuspended in MM buffer to an optical density of 0.4 at OD_600_, and acetosyringone was added to a final concentration of 100 mg L^−1^. The culture solution was incubated at room temperature for 2 h. Genes of interest were transferred into the abaxial leaves of 5-week-old plants, by first nicking the leaf on the backside with a needle and then infiltrating the gene-harboring *A. tumefaciens* using a needleless 1-ml syringe. After 5 days, the infiltrated leaves were collected, frozen in liquid nitrogen, and ground into a fine powder for HPLC analysis.

### Subcellular Localization

*In silico* subcellular prediction of localization was performed using the iPSORT (Bannai et al., 2002), WoLF PSORT (Horton et al., 2007), LOCALIZER (Sperschneider et al., 2017), TargetP (Emanuelsson et al., 2000), and ChloroP servers (Emanuelsson et al., 1999).

For *in planta* experiments on subcellular localization, four biological replicates were used. First, *HaHQT*s were amplified with Phusion Hot Start II High-Fidelity DNA Polymerase (Thermo Fisher Scientific, USA) using pCR™8/GW/TOPO®-*HaHQT*s as templates. The primers (excluding stop codons) listed in Supplementary Table S1 were used in the PCRs. The PCR products were cloned into pCR™8/GW/TOPO®TA vectors (Invitrogen, USA), and nucleotide sequences were verified. Then, the *HaHQT*s were transferred into the C-terminal green fluorescent protein (GFP)-fused destination vector pGWB5 (Nakagawa et al., 2007) using Gateway® LR Clonase® II (Invitrogen, USA), generating pGWB5-*HaHQT*s. The pGWB5-*HaHQT*s were then transformed into *A. tumefaciens* LBA4404 by electroporation.

*A. tumefaciens* containing each construct and *A. tumefaciens* containing a silencing suppressor *p19* gene (Lindbo, 2007) were co-infiltrated into 5-week-old plants (section Plant Material and Growth Conditions) as in section Transient Overexpression of *HaHQT*s in *N. benthamiana* but with some modifications. In brief, cells obtained from each culture were washed, suspended in MM buffer, and adjusted to an optical density of 0.8 at OD_600_. The culture suspensions of each *A. tumefaciens* harboring pGWB5-*HaHQT* construct were then mixed with that of *A. tumefaciens* harboring *p19* at a ratio of 1:1. Then, acetosyringone was added to a final concentration of 100 mg L^−1^. At 3 days after infiltration, protein localization was visualized under FluoView® FV10i-DOC confocal laser scanning microscope (Olympus, Japan). Excitation/emission of GFP, autofluorescence of chloroplast, and phase contrast detection were recorded at 473/510, 559/600, and 559/600 nm, respectively.

### Statistical Analyses

Statistical analyses were performed using IBM® SPSS® Version 22.0 (IBM, USA) statistical software. Following one-way ANOVA, mean concentrations of CQAs and expression levels of genes were compared between days for each CQA or gene type using Duncan’s multiple-range test (*p* < 0.05). In addition, concentration of each CQA and expression levels of genes were compared between hypocotyl and cotyledon tissue types by Student’s *t* test (*p* < 0.05).

## Results

### Caffeoylquinic Acids Profiling in Sunflower Sprouts

**Figure 3:**
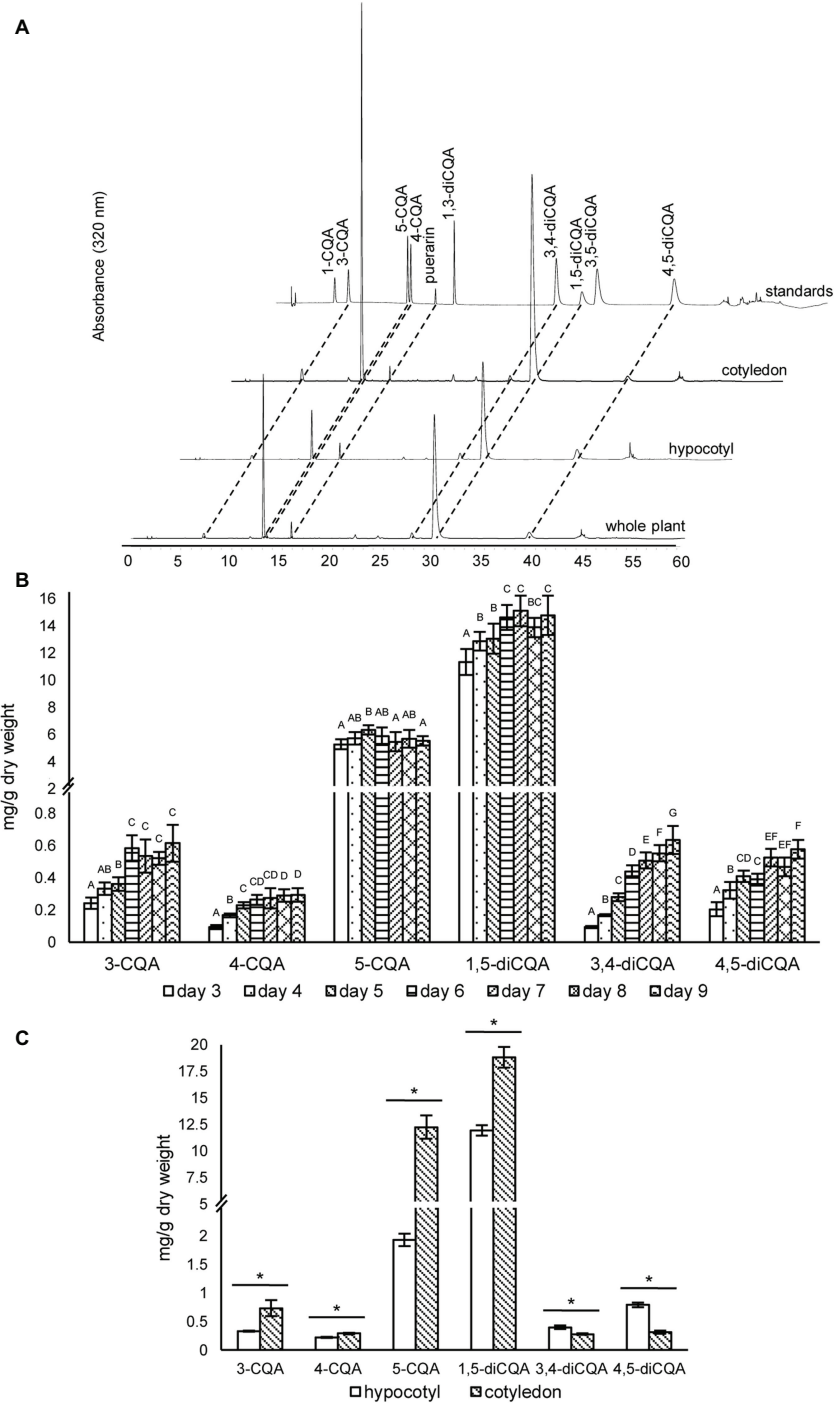
HPLC profiles and concentrations of caffeoylquinic acids in sunflower sprout extracts from different time points during germination and from different tissues. HPLC chromatograms of caffeoylquinic acids of sunflower extracts from whole sprout, hypocotyl, and cotyledon, using puerarin as an internal standard **(A)**; extracts of sunflower sprouts sampled at different time points (days 3–9) during the germination period were measured by HPLC; comparisons are shown for different time points within each caffeoylquinic acid derivative. Bars represent the mean values ± standard deviation (SD) of five biological and independent replicates; for each derivative, different alphabets indicate significant differences according to Duncan’s multiple-range test (*p* < 0.05) **(B)**; and tissue specific concentrations of caffeoylquinic acid derivatives measured in the hypocotyls and cotyledons of sunflower sprouts sampled at 5 day post-germination; an asterisk (∗) above the bars indicates a significant difference between the two tissues (Student’s *t* test, *p* < 0.05) **(C)**.

Although CQAs in sunflower seeds have been reported (Weisz et al., 2009), previous studies have, to the best of our knowledge, not clearly quantified and characterized CQA content in sunflower sprouts. Therefore, we analyzed CQAs during germination from days 3 to 9. Six CQAs were identified (Figure 3A), and 1,5-diCQA was the most abundant. Accumulation of 1,5-diCOA increased during germination, reaching a maximum of ~15 mg/g dry weight (Figure 3B). This increasing accumulation level during sprouting was also observed in other CQAs, including 3-CQA, 4-CQA, 3,4-diCQA, and 4,5-diCQA. Notably, the amount of the second most abundant derivative, 5-CQA, did not significantly change over the period. In addition, at day 5 post-germination, we profiled CQA content in two sunflower sprout tissue types: hypocotyl and cotyledon. Cotyledons accumulated much higher levels of 5-CQA and 1,5-diCQA than hypocotyls and contained ~6-fold higher concentrations of 5-CQA. The other CQAs were detected at much lower levels in both tissues. The identities of all CQAs were also confirmed using LC–MS to compare their fragmentation patterns and molecular masses with those of authentic standards (Supplementary Figure S3).

### Genome-Wide Identification and Phylogenetic Analysis of *HaHQT*s and *HaHCT*s

**Figure 4:**
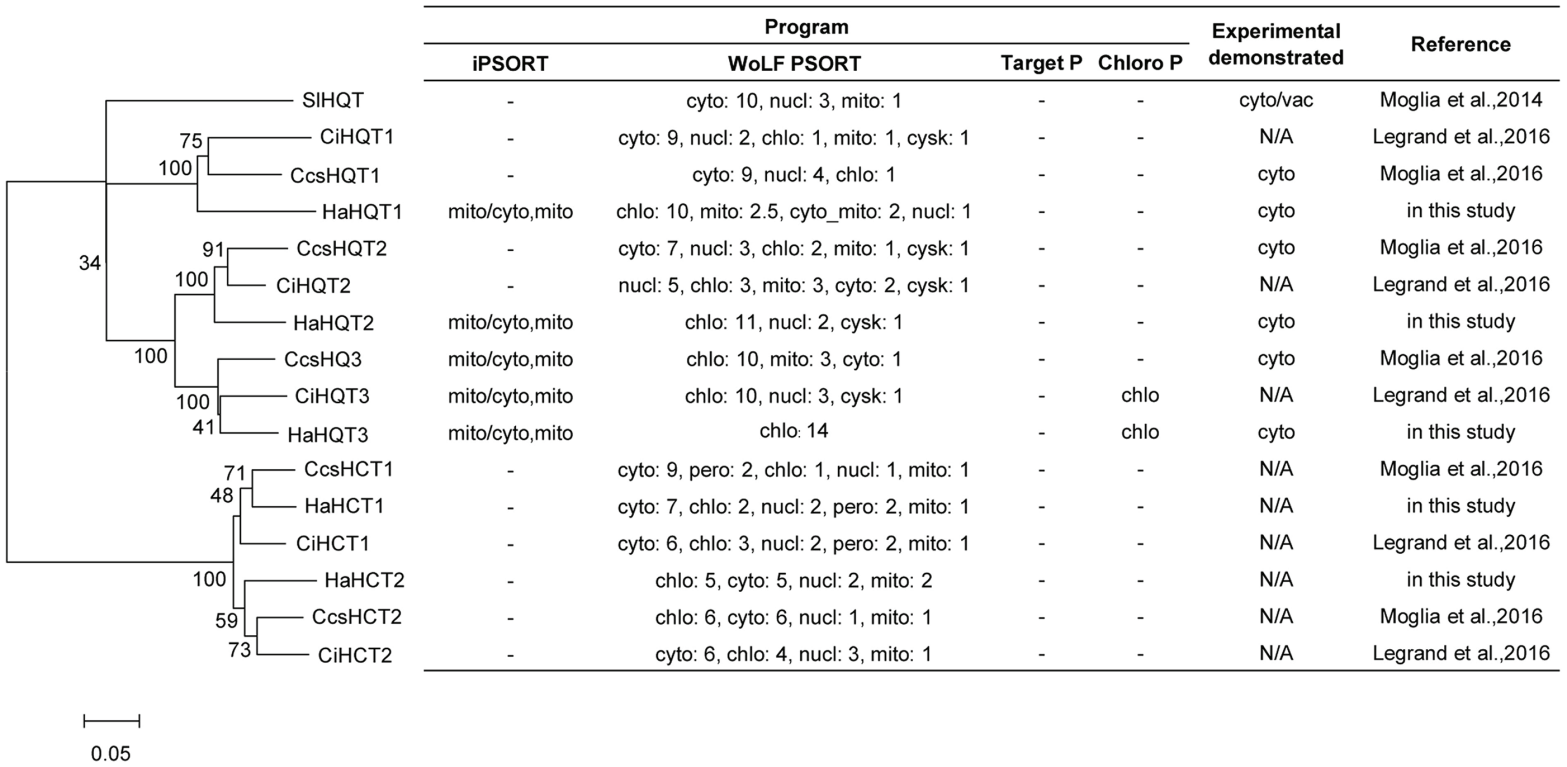
Phylogenetic analysis and *in silico* subcellular localization prediction of sunflower HQTs and HCTs. Sunflower HQTs and HCTs were aligned with HQTs and HCTs from other representative species, including globe artichoke (CcsHQT1; ABK79689.1, CcsHQT2; ADL62854.1, CcsHQT3; ADL62855.1, CcsHCT1; AAZ80046.1, CcsHCT2; KVH99042.1), chicory (CiHQT1; ANN12610.1, CiHQT2; ANN12611.1, CiHQT3; ANN12612.1, CiHCT1; ANN12608.1, CiHCT2; ANN12609.1), and tomato (SlHQT; NP_001234850.2). The phylogenetic tree was constructed with MEGA7 and the neighbor-joining method, using a bootstrap test of phylogeny. *In silico* subcellular localization was predicted using web-based tools, including iPSORT, WoLF PSORT, TargetP, and ChloroP (cyto, cytosol; nucl, nucleus; mito, mitochondria; chlo, chloroplast; cysk, cytoskeleton).

A total of five genes encoding sunflower HQTs and HCTs were identified. Multiple alignment of amino acid sequences of all identified HQTs and HCTs showed the conserved motifs of HXXXD and DFGWG, which are signature to the members of the BAHD superfamily (Supplementary Figure S2). Phylogenetic analysis revealed that three HaHQTs and two HaHCTs were clustered together with HQTs and HCTs from chicory and globe artichoke (Figure 4). The number of HQT and HCT isoforms found in sunflower was identical to the number identified previously in chicory and globe artichoke. We annotated HaHQTs (HaHQT1, HaHQT2, and HaHQT3) and HaHCTs (HaHCT1 and HaHCT2) based on their clustering with the previously characterized HQTs and HCTs from those two species. Moreover, these *HaHQT*s and *HaHCT*s were located on different chromosomes, e.g., *HaHQT1* on chromosome 9, *HaHQT2* on chromosome 10, *HaHQT3* on chromosome 2, *HaHCT1* on chromosome 16, and *HaHCT2* on chromosome 5.

### Gene Expression Profiles of *HaHQT*s and *HaHCT*s in Sunflower Sprouts

**Figure 5:**
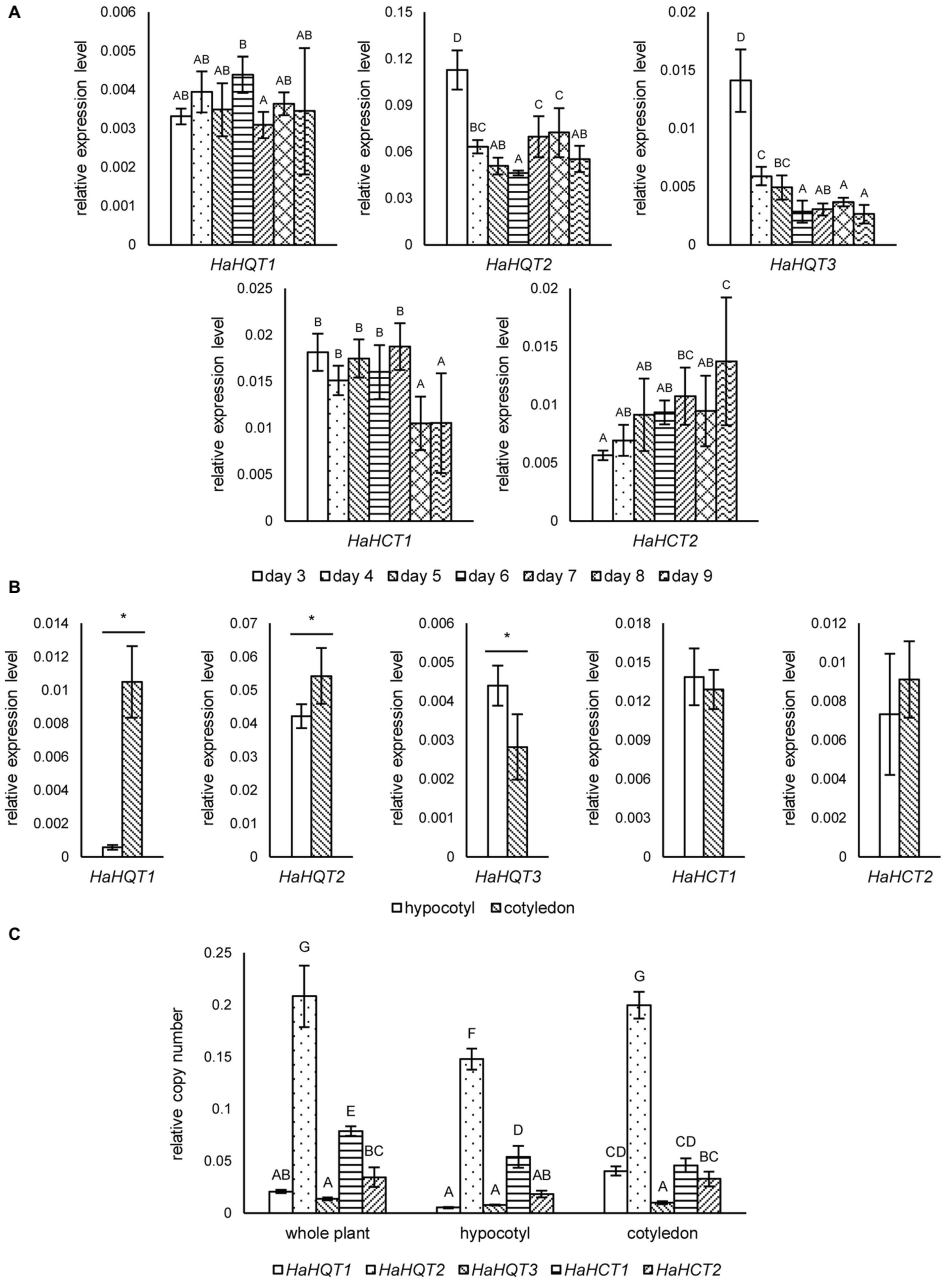
qRT-PCR analysis of sunflower *HQT*s and *HCT*s at different germination time points and tissues. Relative expression levels of sunflower *HQT*s (*HaHQT*s) and *HCT*s (*HaHCT*s) measured at different time points (days 3–9) during the germination period. Bars represent the mean values ± standard deviation (SD) of five biologically independent replicates; comparisons are shown among different time points within each gene; different alphabets indicate significant differences according to Duncan’s multiple-range test (*p* < 0.05) **(A)**. Tissue specific gene expression analysis of *HaHQT*s and *HaHCT*s sampled at day 5 post-germination; for each gene, comparisons are shown between the hypocotyl and cotyledon; an asterisk (*) above the bars indicates a significant difference between the two tissues (Student’s *t* test, *p* < 0.05) **(B)**. Absolute gene expression analysis of *HaHQTs* and *HaHCTs* from whole plant, hypocotyl, and cotyledon using digital droplet PCR (ddPCR). Bars represent the mean values ± standard deviation (SD) of five biologically independent replicates; comparisons are shown for all genes among different tissues; different alphabets indicate significant differences according to Duncan’s multiple-range test (*p* < 0.05) **(C)**.

To investigate a possible correlation between CQA content and expression levels of corresponding biosynthetic genes, qRT-PCR was used to analyze the expression profiles of identified *HaHQT*s and *HaHCT*s. Sunflower sprouts for this analysis were sampled at seven time points, from days 3 to 9 after germination. As shown in Figure 5A, expression level of *HaHQT1* was not that much different among these time points. However, the expression level of *HaHQT2* was peaked at day 3 and was followed by a significant decrease from day 4. Similarly, expression level of *HaHQT3* was peaked at day 3 and was decreased until day 9, whereas expression level of *HaHCT1* was constant from days 3 to 7 and was significantly decreased from days 7 to 8. Then, it was kept constant again. Noticeably, expression level of *HaHCT2* was increased during germination period. In addition, we investigated the expression levels of these genes in hypocotyls and cotyledons at day 5. Interestingly, *HaHQT1* and *HaHQT2* were expressed at significantly higher levels in cotyledons than in hypocotyls, while that of *HaHQT3* was significantly higher in hypocotyl than in cotyledon (Figure 5B). However, the expression levels of two *HaHCT*s were not significantly different between the two tissues. Gene expression analysis by ddPCR confirmed the higher expression level of *HaHQT2* than other *HaHQT*s and *HaHCT*s (Figure 5C). These results provided compelling evidence that *HaHQT2* could be the main CQA biosynthetic gene in the sunflower sprouts. Therefore, we selected *HaHQT*s for further functional characterization.

### Promoter Analysis

To gain more insights into the regulatory network controlling the expression levels of HaHQT genes, we analyzed the promoter regions of those HaHQT genes. Due to the incomplete genome database, only promoter regions of HaHQT2 and HaHQT3 were analyzed. As shown in Supplementary Table S2, these two promoters share some common motifs such as phytochrome, defense response, circadian rhythm, axillary bud outgrowth, light, stress, and phytohormone (auxin, salicylic acid, abscisic acid, jasmonate, gibberellin, ethylene, cytokinin) responsive elements as well as sulfur and sucrose responsive elements. In addition, we found several binding elements for MYB and Dof transcription factors. These two transcription factors were reported to be involved in the phenylpropanoid biosynthesis.

### Subcellular Localization of HaHQTs

**Figure 6:**
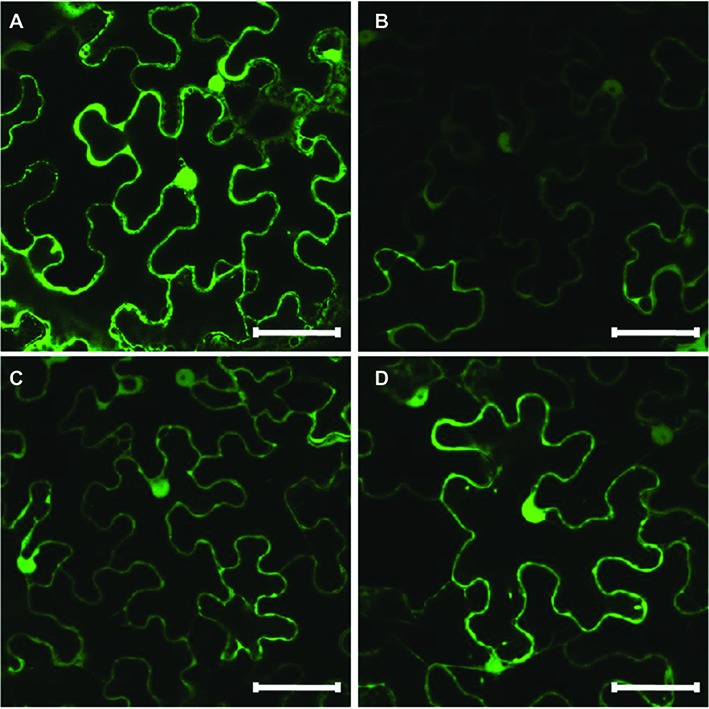
Subcellular localization of GFP-tagged HaHQTs in the epidermis of *Nicotiana benthamiana* leaves. Confocal microscopy image of epidermal cells from *N. benthamiana* leaves infiltrated with pGWB2-*GFP* (control) **(A)**, pGWB5-*HaHQT1* **(B)**, pGWB5-*HaHQT2* **(C)**, or pGWB5-*HaHQT3* **(D)**. Bars = 50 μm.

*In silico* subcellular analysis of sunflower HaHQTs predicted their localization in either cytosol or chloroplast (Figure 4) with no detection of nuclear localization signal (NLS) in all HaHQTs. For assessment *in planta*, *Agrobacteria* harboring each GFP-fused expression construct (pGWB5-*HaHQT1*, pGWB5-*HaHQT2*, or pGWB5-*HaHQT3*) together with the gene-silencing suppressor *p19* were infiltrated in *N. benthamiana* leaves. Protein localization was analyzed using a confocal laser scanning microscope. *In planta*, all three GFP-tagged HaHQTs were soluble and not membrane-bound proteins, possibly localized in the cytosol (Figure 6; Supplementary Figure S4). Fluorescence signals were also detected in the nucleus. In addition, observation of mesophyll clearly confirmed that these HaHQTs were not localized in chloroplast (Supplementary Figure S5).

### Transient Expression of *HaHQT*s in *Nicotiana benthamiana*

**Figure 7:**
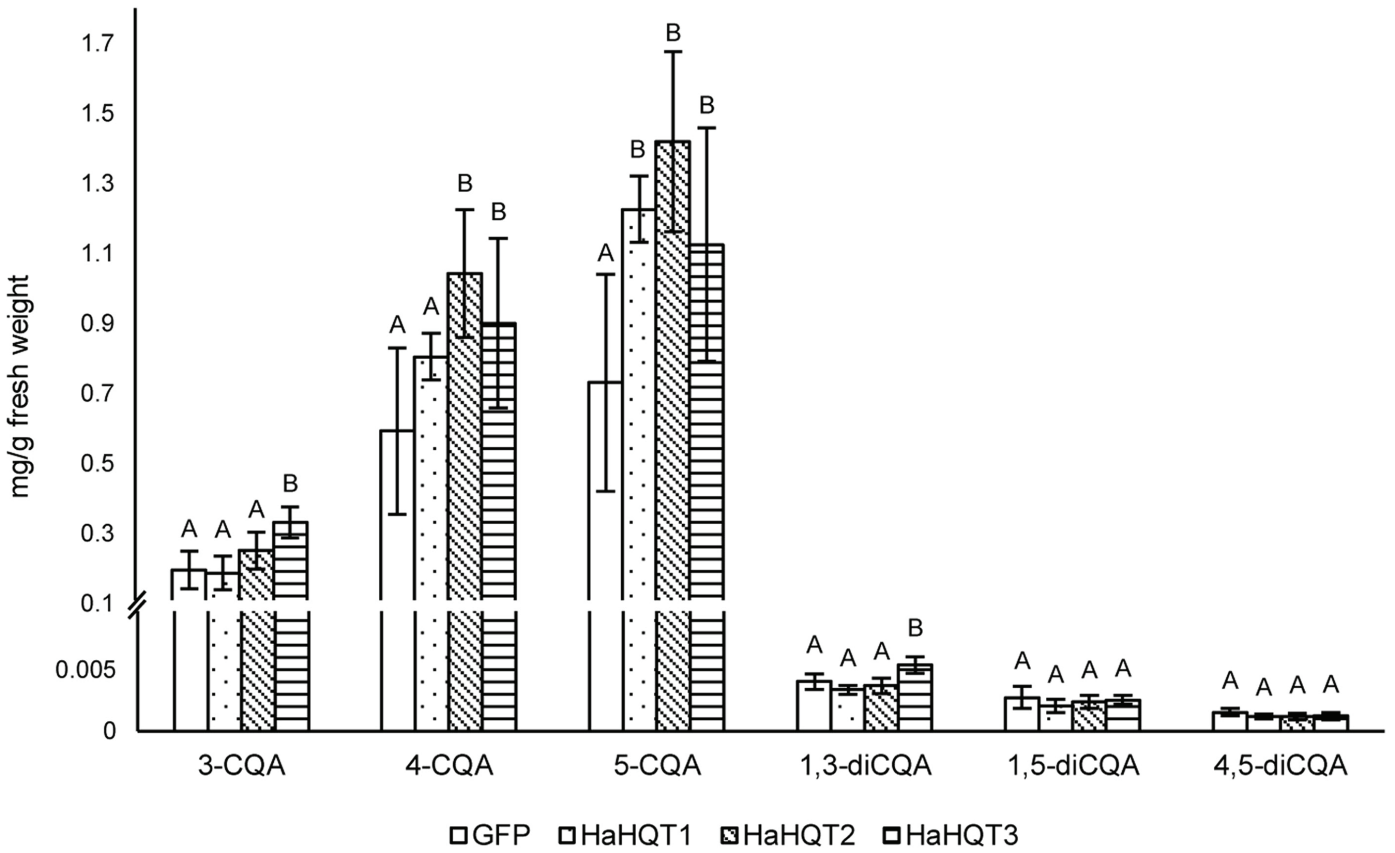
HPLC profiling of caffeoylquinic acids in *Nicotiana benthamiana* leaves infiltrated with *HaHQT*s. Tissue extracts of *N. benthamiana* leaves infiltrated with *GFP* (control) and *HQT*s from sunflower (*HaHQT1*, *HaHQT2*, and *HaHQT3*) were analyzed by HPLC. Bars represent the mean values ± standard deviation (SD) of five biologically independent replicates. Within each caffeoylquinic acid derivative, different alphabets indicate significant differences according to Duncan’s multiple-range test (*p* < 0.05).

In *N. benthamiana* leaves infiltrated to transiently express *HaHQT*s, levels of 5-CQA were significantly higher than the control (Figure 7). *HaHQT2* and *HaHQT3* also increased the level of 4-CQA. *HaHQT3* is the only isoform that could increase the level of all monoCQAs and 1,3-diCQA. The levels of 1,5-diCQA and 4,5-diCQA did not show any significant differences between the infiltrated leaves versus the control. These results indicated that all *HaHQT*s were involved in CQA biosynthesis.

## Discussion

Mono and diCQAs are known to be beneficial to human health. The monoCQA 5-CQA, also known as chlorogenic acid, has a wide variety of health benefits (see review Naveed et al., 2018) and is one of the most abundant CQAs. Although sunflower seed kernels accumulate much higher amounts of monoCQAs than diCQAs (Weisz et al., 2009), we found significantly higher content of diCQAs than of monoCQAs in sunflower sprouts. Our results concur with those of Sun et al. (2012) in which 1,5-diCQA is the most abundant CQA in sprouts (Figure 3B). The lower level of monoCQAs found in sprouts could be related to the role of monoCQAs as intermediates used for both diCQA biosynthesis and lignification in growing hypocotyls during sprouting (Escamilla-Treviño et al., 2014). Although 3-CQA, 4-CQA, 1,5-diCQA, 3,4-diCQA, and 4,5-diCQA contents tended to increase in sunflower sprouts until day 9 (Figure 3B), we did not analyze their content after day 9. This was because sunflower sprouts available in markets are generally harvested at day 5 or 6, when the average hypocotyl length is 12–15 cm (Supplementary Figure S1B). Therefore, we designed our experiment to include sampling shortly before (days 3–4) and after (days 7–9) the common harvesting period. Harvest after day 9 would negatively affect the texture of the sprouts, *e.g.* they would be less crisp, which is inconsistent with consumer preferences. Among CQAs, only 5-CQA did not increase in concentration during the germination period, perhaps because it was being converted to 1,5-diCQA or used for lignin biosynthesis (Figure 2). Competing use of 5-CQA for lignin biosynthesis versus production of 1,5-diCQA during sprouting could contribute to the less abundance of monoCQAs than diCQAs in sprouts. Regarding the distribution of CQAs in hypocotyls and cotyledons, higher amounts of CQAs in cotyledons (Figure 3C) were probably because the role of these compounds is in protecting against herbivores, pathogens, and harmful UV light to which cotyledons are exposed to in nature (Niggeweg et al., 2004; Cle et al., 2008; Leiss et al., 2009).

We performed a genome-wide analysis to increase our understanding of CQA biosynthesis and discovered three *HaHQT*s and two *HaHCT*s. These enzymes were well clustered with the previously characterized *HaHQT*s and *HaHCT*s from chicory and globe artichoke (Figure 4). These results demonstrated close evolutionary relationships and some level of domain consensus among different HQT and HCT isoforms between sunflower and other Asteraceae family plants (chicory and globe artichoke). Although predictions from our *in silico* analysis by ChloroP suggested HaHQT3 would be localized to chloroplasts (Figure 4), we found that all HaHQTs fused with GFP localized in the cytoplasm (Figure 6); but we also observed fluorescence signals in the nucleus. This may result from protein diffusion into the nucleus, consistent with the previous characterization of HQT2 from globe artichoke (Moglia et al., 2016). A nucleo-cytoplasmic localization was in addition documented for other members of the BAHD superfamily, namely spermidine hydroxycinnamoyl transferases (Delporte et al., 2018).

Both HQTs and HCTs are involved in biosynthesis of CQAs in chicory (Legrand et al., 2016). Our expression analysis showed that *HaHQT2* was expressed at a dramatically higher level than other *HQT*s and *HCT*s in both hypocotyl and cotyledon tissues (Figure 5C). In addition, in cotyledons, the higher expression of *HaHQT*2 coincided with higher content of CQAs than that in hypocotyls (Figures 5B,C). This suggests that, rather than the other genes, HaHQT2 could be the main enzyme involved in CQA biosynthesis during sunflower sprout germination. Studies in tomato (Niggeweg et al., 2004), potato (Payyavula et al., 2015), globe artichoke (Moglia et al., 2016), and Japanese honeysuckle (*Lonicera japonica*; Zhang et al., 2017) reached a similar conclusion that HQTs have an important role in CQA biosynthesis. By contrast, based on functional characterization of *N. benthamiana*, Legrand et al. (2016) showed that HCT1 from chicory played a major role in CQA biosynthesis. These results suggest that the mechanisms of CQA biosynthesis regulation could vary between closely related plant species.

To gain a better understanding of factors controlling the expression of *HaHQT*s, we searched the promoter regions of *HaHQT2* and *HaHQT3* for relevant regulatory elements. Multiple elements associated with stress response and hormonal signaling were found in the promoters of both genes. In addition, several MYB and Dof regulator elements were also found in the promoter regions of both genes. MYB transcription factor has been known to regulate CQA biosynthesis. Transient overexpression of MYB1 from eggplant (*Solanum melongena*) in *N. benthamiana* could enhance the accumulation level of 5-CQA (Docimo et al., 2016). Moreover, 5-CQA content was significantly increased in leaves and fruit of transgenic tomato lines overexpressing Arabidopsis MYB12 (Pandey et al., 2015). The involvement of Dof transcription factor in phenylpropanoid biosynthesis has been reported. Arabidopsis Dof4.2 negatively affected flavonoid biosynthesis but positively regulated hydroxycinnamic acid biosynthesis (Skirycz et al., 2007). Since sunflower sprouts accumulate comparably high levels of CQAs, this sprout might be suitable for further identification of novel transcription factors controlling CQA biosynthesis. Nevertheless, the different expression levels of those two *HaHQT*s might be due to other regulating factors such as different positions/numbers of the cis-acting elements in the promoter regions. Further *in planta* functional analysis confirmed the role of all HaHQTs in CQA biosynthesis. Compared with control *N. benthamiana* plants infiltrated with *GFP*, leaves infiltrated with *HaHQT*s had higher 5-CQA content, and the highest increase (94%) occurred in leaves infiltrated by *HaHQT2* (Figure 7). These results suggest that *HaHQT*s are involved in the biosynthesis of 5-CQA, the most abundant monoCQA found in sunflower sprouts. Consistently, in *N. benthamiana* leaves infiltrated with artichoke *HQT*s, among other monoCQAs, 5-CQA content was mainly affected (Moglia et al., 2016). As for diCQAs, only 1,3-diCQA content was increased slightly by the infiltration of *N. benthamiana* leaves with *HaHQT3* construct. This observation was different from a study by Moglia et al. (2016) which found that *N. benthamiana* leaves infiltrated with artichoke HQTs had increased diCQA contents at much higher level than that of *HaHQT3-*transiently expressed *N. benthamiana* leaves. Because of this, we hypothesized that the increased amount of diCQA in *N. benthamiana* leaves infiltrated with artichoke HQTs might be due to the dramatically increased level of monoCQAs (up to 500% for 5-CQA; Moglia et al., 2016), as diCQAs are synthesized from monoCQAs. However, in our study, we did not observe as high an amount of 3-CQA in the *HaHQT*s-infiltrated *N. benthamiana* leaves as Moglia et al. (2016) did, which might explain why we did not observe an increase in diCQA contents. In a study by Legrand et al. (2016), infiltration of *N. benthamiana* leaves with either *HQT1* or *HCT1* from chicory did not lead to a great increase in 3-CQA content (only up to ~19 and ~56%, respectively), which is similar to results from our study. Unfortunately, Legrand et al. (2016) did not mention any changes occurring in diCQA contents. Differences in the expression vectors used and in the growth stages and conditions of *N. benthamiana* between studies may partially explain why production of monoCQAs in *N. benthamiana* infiltrated with *HQT*s from different sources is variable. In addition, there may be differences in the catalytic efficiency of HQTs investigated in different studies, and this possibility should be investigated further.

Although sunflower sprouts accumulate much higher level of 1,5-diCQA than globe artichoke and chicory (Pandino et al., 2011; Willeman et al., 2014), our results did not provide a strong evidence to support the role of HaHQTs and HaHCTs in diCQA biosynthesis. Therefore, mechanisms behind biosynthesis of diCQAs remain unclear. It is possible that other acyltransferases might be involved in diCQA biosynthesis. Since the sunflower genome has recently been reported (Badouin et al., 2017), identification of additional acyltransferases in the genome, together with transcriptome analysis, might help in identifying novel candidate genes involved in diCQA production. So far, the only HQT enzyme known to produce both monoCQAs and diCQAs is from tomato, where it is localized to both the cytosol and vacuoles (Moglia et al., 2014). The amino acid residue His276 has been identified as partially responsible for the dual function of tomato HQT and mutation of this residue to Tyr decreases the production of diCQA *in vitro*. In chicory, globe artichoke, and sunflower, a Tyr residue is found at this position (Supplementary Figure S2). Therefore, it is unlikely that HQTs in these three species would have the dual function seen in tomato HQT.

In conclusion, we have reported biosynthesis of CQAs in sunflower sprouts for the first time. Sunflower sprouts are a rich source of monoCQAs and diCQAs, and HaHQT2 was found to be the major isoform, which could be responsible for CQA biosynthesis during germination in both hypocotyls and cotyledons. Therefore, manipulation of this gene could positively affect CQA contents in sunflower sprouts. Thus, our results provide informative data, which could be applied to further biofortify sunflower sprouts as functional foods.

## Data Availability

The raw data supporting the conclusions of this manuscript will be made available by the authors, without undue reservation, to any qualified researcher.

## Author Contributions

SS conceived the research. KC performed most of the experiments and analyzed the data. PB participated in genome-wide identification. KC, GK, PP, and SS interpreted the data and drafted the manuscript. All authors have read and approved the final manuscript.

### Conflict of Interest Statement

The authors declare that the research was conducted in the absence of any commercial or financial relationships that could be construed as a potential conflict of interest.

## Footnotes

^1^www.sunflowergenome.org
