# Supplementary material for: Identification and Functional Characterization of Genes Involved in the Biosynthesis of Caffeoylquinic Acids in Sunflower (Helianthus annuus L.)
Source: Front Plant Sci. 2019 Jul 31;10:968. doi: 10.3389/fpls.2019.00968 (PMC6685037; doi:10.3389/fpls.2019.00968)
Supplement: Supplementary file 1 [file Data_Sheet_1.pdf]

Supplementary Material

Supplementary Material

A

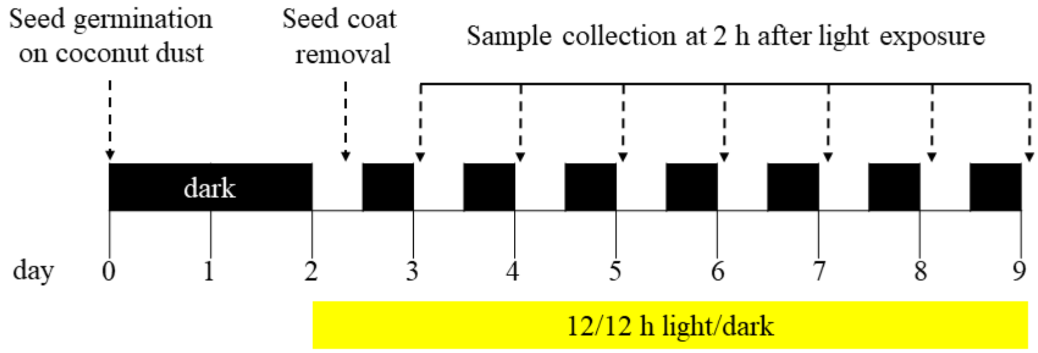

B

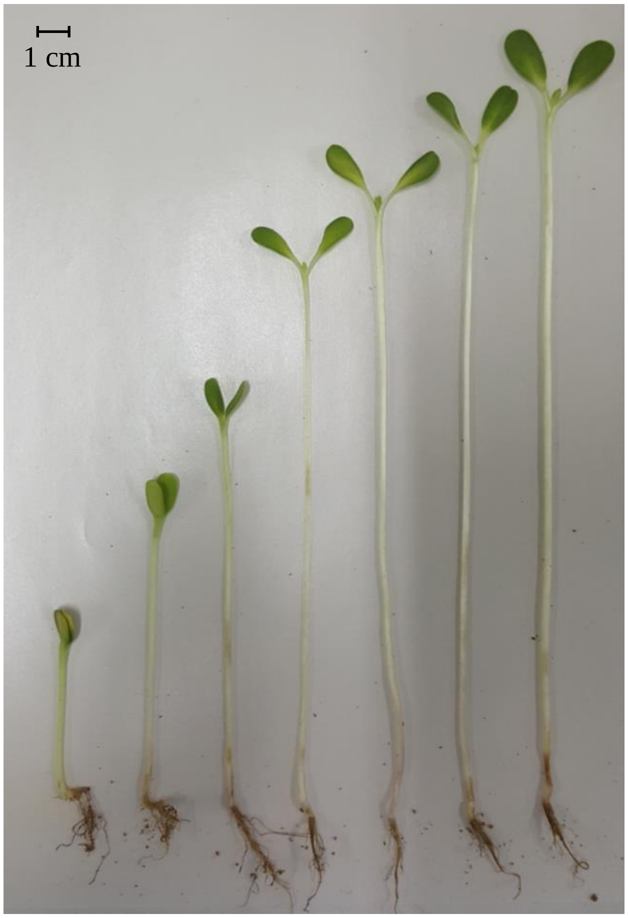

Supplementary FIGURE S1 | Sunflower sprouts used in this study. Schematic diagram of sampling in this study **(A)** and the representative sunflower sprouts were imaged at different timepoints (day 3 to day 9, from left to right respectively) during their germination period **(B)**.

```
SlHQT        -MG--SEKMMKINIKESTLVKPSKPTPT--KRIWSSNLDLIVGRIHLLTVYFYKPNGSSN   55
CcsHQT1      ---------MELTVKESLMVKPSKPTPN--QRLWNSNLDLVVGRIHILTVYFYRPNGSSN   49
CcsHQT2      -MGSDQKMMMNIDIMKSSIVPPSELIADCPKQLWTSNLDLVVGRIHILTVYFYRPNGSSK   59
CcsHQT3      ---------MNINIKHSSFVQPSQPTPS--STIWTSNLDLVVGRIHILTVYFYRPNGASN   49
CcsHCT1      ---------MKIEVRESTMVRPAEETPR--INLWNSNVDLVVPNFHTPSVYFYRPNGAAN   49
CcsHCT2      ---------MKIAVRESTMVRPAEETPM--IKLWNSNVDLVVPNFHTPSVYFYRPTGAGN   49
CiHQT1       MTNGAGSEKMKVTVKESVIVKPSKTTPI--QRLWNSNIDLLVGRIHLLTIYFYRPNGSSD   58
CiHQT2       -MRSDQKMMMNINLKKSSIIPPSETIAECPKQLWTSNLDLVVGRIHILTVYFYRPNGSAN   59
CiHQT3       -MGSD-HKTMKINIKQSSLVQPSKPTIPSNKKLWTSNLDLVVGRIHILTVYFYRPNGCSN   58
CiHCT1       ---------MKIEVRESTMVRPAEETPK--INLWNSNVDLVVPNFHTPSVYFYRPNGATN   49
CiHCT2       ---------MKIAIRESTMVRPAEETPR--IKLWNSNVDLVVPNFHTPSVYFYRPTGATN   49
HaHQT1       ---------MNLTVKQSLTIKPSKTTPC--HTIWCSNLDLIVGRIHIQTVYFYRPNGSSN   49
HaHQT2       -MKTDQTKPMNITITKSSIVPPSETINGSSNHIWTSNLDLVVGRIHILTVYFYRPNGSNN   59
HaHQT3       -MGTV-QKTLNINIKQTTFVQPSKPTQASTKQLWTSNLDLVVGRIHILTVYFYRPIVCSN   58
HaHCT1       ---------MKIEVRESTMVKPAEETPK--INLWNSNVDLVVPNFHTPSVYFYRPNGAPD   49
HaHCT2       ---------MKITVTNSSMVRPAEETPR--IKLWNSNVDLVVPNFHTPSVYFYRPNGGSN   49
                      ::: : .:  : *::        :* **:**:* .:*  ::***:*    .
SlHQT        FFDNKVIKEALSNVLVSFYPMAGRLGRDEQGRIEVNCNGEGVLFVEAESDSCVDDFGDFT   115
CcsHQT1      FFDSGVLKKALADVLVSFFPMAGRLGNDGDGRVEINCNGEGVLFVEAEADCSIDDFGEIT   109
CcsHQT2      FFDPNVMKKALADVLVSFYPMAGRLGRDETDRIVINCNNEGVLFVEAESDSTLDDFGELK   119
CcsHQT3      FFDADVMKKALADVLVSFYPMAGRISRDRNGRLEINCNGEGVLFVEAELDSTLDDFGEFT   109
CcsHCT1      FFDPKVMKDALSRALVPFYPMGGRLKRDEDGRIEIDCQGQGVLFVEAESDGVIDDFGDFA   109
CcsHCT2      FFDPTVMKDALSRVLVPFYPMGGRLSRDEDGRIEIDCRGQGVLFVEAESDGMIDDFGDFA   109
CiHQT1       FFDSGVLKKALSDVLVSFFPMAGRLGKDGDGRVEINCNAEGVLFVEAEADCRIDDFGEIT   118
CiHQT2       FFDPNVMKKALADVLVSFYPMAGRLDRDESGRIVINCNGEGVLFVEAESDSTLDDFGEFT   119
CiHQT3       FFDPVVMKKALADVIVSFYPFAGRLSRDQNGRLEINCNGEGVLFVEAESDSTLDDFGEFT   118
CiHCT1       FFDPKVMKDALSKALVPFYPMGGRLKRDEDGRIEIDCQGQGVLFVEAESDGMVDDFGDFA   109
CiHCT2       FFDAKVMKDALSRALVPFYPMGGRLKRDEDGRIEIDCQGQGVLFVEAESDGVIDDYGDFA   109
HaHQT1       FFDSEVLKAALADVLVSFYPMSGRLGKDGDGRIEIVCNGEGVLFVEAEADCCIDDFGEIT   109
HaHQT2       YFDPNVMKQALADVLVPFYPMAGRLGRDESGRIVINCNGEGALFVEAESDSCLDDFGEFT   119
HaHQT3       FFDPLVMKKALADVLVTFYPMAGRMGKDQNGKVVINCNDEGVLFVEAESDSTLDDFGEFT   118
HaHCT1       FFDPKVMKDALSRALVPFYPMGGRLKRDEDGRIEIDCRGQGVLFVEAESDGVVDDFGDFA   109
HaHCT2       FFDPKVMIKALSKALVPFYPMGGRLTRDEDGRIEIDCQGQGVLFVEAESDGVIDDFGDFA   109
             :**  *:  **: .:* *:*:.**: .*  .:: : *. :*.****** *  :**:*::
SlHQT        PSLELRKLIPSVETSGDISTFPLVIF------QITRFKCGGVALGGGVFHTLSDGLSSIH   169
CcsHQT1      PSPELRKLAPTVDYSDQVSSYPLCIT------QVTRFNCGGVSLGCGLHHTLSDGLSSLH   163
CcsHQT2      PSPVFRQLTPSVDYSGDISSYPLLFA------QVTHFKCGGVALGCGVHHTLSDGLSLLH   173
CcsHQT3      PSPELRRLTPTVDYSGDISSYPLFFA------QVTHFKCGGVALGCGVFHALADGLSSIH   163
CcsHCT1      PTLELRKLIPAVDYTLGIESYSLLVL------QVTYFKCGGVSLGVGMQHHAADGASGLH   163
CcsHCT2      PTLELRKLIPAVDYSLGIESYSLLVL------QVTYFKCGGVSLGVGMQHHAADGASGLH   163
CiHQT1       PSPELRRLAPTVDYSGDISSYPLFIT------QVTRFKCGGVSLGCGLHHTLSDGFSSLH   172
CiHQT2       PSPEFRRLTPTVDYSGDISSYPLFFA------QVTHFKCGGVALGCGVFHTLSDGLSSLH   173
CiHQT3       PSPELRSLTPTVDYSGDISSYPLFFA------QVTHFKCGGVGLGCGVFHTLADGLSSIH   172
CiHCT1       PTLELRKLIPAVDYSQGIESYPLLVL------QVTYFKCGGVSLGVGMQHHAADGASGLH   163
CiHCT2       PTLELRKLIPAVDYSLGIESYSLLVL------QVTYFKCGGVSLGVGMQHHAADGASGLH   163
HaHQT1       PSPELGRLAPTVDYSGDISSYPLFVT------QVTRFKCGGVSLGSALHHTLADGLSALH   163
HaHQT2       PSPEFRSLTPTVDYSGDISSFPLFFA------QVTHFKCGGVALGCGVFHTLSDGLSSLH   173
HaHQT3       PSPELRRLTPTVDYSGDISSYPLFFAQVYWFDQVTHFKCGGVGLGCGVFHTLADGLSSLH   178
HaHCT1       PTLELRKLIPAVDYSQGIESYPLLVL------QVFNKFCGGVSLGVGMQHHAADGASGLH   163
HaHCT2       PTLELRKLIPVVDYTLGIGSYPLLVL------QVTYFKCGGVSLGVGMQHHAADGASGLH   163
             *:  :  * * *: :  : :: * .       *:    ****.** .: *  :** * :*
```

Supplementary FIGURE S2 | Multiple sequence alignment of HQTs and HCTs from sunflower (HaHQTs and HaHCTs) and other representative species. Amino acid sequences of HaHQTs and HaHCTs are shown and aligned with those from other representative species including tomato (SlHQT; NP_001234850.2), globe artichoke (CcsHQT1; ABK79689.1, CcsHQT2; ADL62854.1, CcsHQT3; ADL62855.1, CcsHCT1; AAZ80046.1, and CcsHCT2; KVH99042.1), and chicory (CiHQT1; ANN12610.1, CiHQT2; ANN12611.1, CiHQT3; ANN12612.1, CiHCT1; ANN12608.1, and CiHCT2; ANN12609.1).

```
SlHQT       FINTWSDIARGLSVAVPPFIDRTLLRARDPPTSSFEHVEYHPPPTLNSSKNR--------  221
CcsHQT1     FINTWSDKARGLSVAIPPFIDRSLLRARDPPTAMFEHLEYHSPPSLIAPSQNQNF-TSHP  222
CcsHQT2     FINTWSDMARGLSVAIPPFIERTLLRAREPPTPTYDHVEYHSPPSMNTTAQKPGS-GSL-  231
CcsHQT3     FINTWSDMARGLSIAIPPFIDRTLLRAREPPTPTFDHIEYHAPPSMKTISQNPES-S---  219
CcsHCT1     FINTWSDLARGLDLAVPPFIDRTLLRSRDPPQPAFDHIEYQPAPPMKTAPTPTPTDDESV  223
CcsHCT2     FINAWSDMARGLDLTLPPFIDRTLLQARDPPVPVFEHVEYQPAPPMKLAPKSA------S  217
CiHQT1      FINTWSDVARGLSVAIPPFNDRTLLRARDPPTPMFDHVEYHPPPSLITPPENHK------  226
CiHQT2      FINTWSDMARGLSVAIPPFIERTLLRAREPPTPTHDHVEYHEPPSMSPTTQKSGS-GS--  230
CiHQT3      FINTWSDMTRGLSIAVPPFTDRTLLRARETPTPTFDHVEYHLPPTMNTTSQNPKS-S---  228
CiHCT1      FINTWSDMARGLDLTIPPFIDRTLLRARDPPQPAFEHVEYQPAPQMKTTS--KSTDEQPV  221
CiHCT2      FINTWSDMARGLDVSLPPFIDRTLLRAQDPPRPVFEHVEYQPAPPMKSTSEST------S  217
HaHQT1      FINTWSDKARGLTVAVPPFVDRTLLRARDPPTPMFDHVEYHQPPSMIAPLENQKS-TSDP  222
HaHQT2      FINTWSDVARGLSVAIPPFIDRTLLRARVPPTPTHDHVEYHPPPTMITTQ---KT-GSL-  228
HaHQT3      FINTWSDMARGLSIAIPPFIDRTLLRAREPPTPTFDHVEYHPPPSMKPDSQKPES-S---  234
HaHCT1      FINTWSDMARGLDITIPPFIDRTLLRARDPPQPVFDHIEYQPAPPMKNAP--TTTNDQPV  221
HaHCT2      FINAWSDMARGLDITLLPFIDRTLLKARDPPQPVFEHVEYQPAPPMKSLPEVE------S  217
            ***:*** :*** ::: ** :*:**:::  *   .:*:**:  * :
SlHQT       ESSTTTMLKFSSEQLGLLKSKSKNEG-----STYEILAAHIWRCTCKARGLPEDQLTKLH  276
CcsHQT1     KLASTAMLRLTLDQINGLKSKAKGDGS-VYHSTYEILAAHLWRCACEARGLSDDQPTKLY  281
CcsHQT2     SKSSTTMLKLTLDQLNSLKAKAKSESG-STHSTYEILAAHIWRCACKARGLPDDQLSKLY  290
CcsHQT3     RKPSTTVLKLTLDQLNVLKASTKNDGSNTTYSTYEILAAHLWRCACKARGLPDDQLTKLY  279
CcsHCT1     PETTVSIFKLTRDQVNALKGKSKEDGNTVNYSSYEMLSGHVWRCVCKARGLPDDQDTKLY  283
CcsHCT2     DETVVSMFKLTRDQLNGLKAKSKEDGNTINYSSYEMLSGHVWRSVCKARGLKDDQDTKLY  277
CiHQT1      PPATTTILRLTLDQINDLKSKGKGDGS-VYHSTFVIIAAHLWRSACKARGLSDDQPTKLY  285
CiHQT2      ---STTMLKLTLDQLNTLKVKAKIESG-QTHSTYEILAAHIWRCACKARGLPDDQLTKLY  286
CiHQT3      RKPSTNMLKLTIDQLNALKTAAKNDGGNTAYSTYEILAAHLWRCACKARGLPNDQQTKLY  288
CiHCT1      PETAVAIFKLTRDQLNALKAKSKENGNTINYSSYEMLSGHVWRCVCKARNLPADQDTKLY  281
CiHCT2      DETVVSMFKLTRDQLNVLKSKSKEAGNTINYSSYEMLSGHVWRSVCKARGLPEDQDSKLY  277
HaHQT1      KSASTMMLRLTLDQINDLKSKAKGDGS-AYHSTYEILAAHIWRCACKARGLSDDQPTKLY  281
HaHQT2      SKSSTTMLKLTLDQLNTLKAKAKSEGG-ASYSTYETLAAHIWRCACKARGLPDDQLTKLY  287
HaHQT3      RKPSTTILKLTLDQLNALKAAAKNDGGNTNYSTYEILAAHLWRCACKARGLPDDQLTKLY  294
HaHCT1      PETAVSIFKLTRDQLNALKAKSKEAGNTISYSSYEMLSGHVWRCVCKARGLPDDQDTKLY  281
HaHCT2      DETVVSIFKLTRDQLNALKSKSKEDGNAINYSSYEMLSGHVWRSVCKARGLTNDHDTKLY  277
                . ::::: :*:. **    *  .      *::  ::.*:**..*:**.*  *: :**:
SlHQT       VATDGRSRLCPPLPPGYLGNVVFTATPIAKSCELQSEPLTNSVKRIHNELIKMDD-NYLR  335
CcsHQT1     VATDGRSRLNPPLPPGYLGNVVFTATPIAKSGEFKSESLADTARRIHSELAKMDD-QYLR  340
CcsHQT2     VATDGRSRLSPRLPPGYLGNVVFTATPVAKSGDLTSKSLSNTAKLIHTTLTKMDD-DYLR  349
CcsHQT3     VATDGRSRLSPQLPPGYLGNVVFTTTPVAKSGDLTTQSLSNAASLIRTTLTKMDN-NYLR  338
CcsHCT1     IATDGRARLRPSLPRGYFGNVIFTTTPIAVAGDLQSKPTWYAASKIHDALARMDD-DYLK  342
CcsHCT2     IATDGRARLQPALPAGYFGNVIFTTTPIAVAGELQSKPTWYAASKIHDALAKMNN-DYLK  336
CiHQT1      VATDGRSRLIPPLPCGYLGNVLFTTTPMAKSGEFKSESLADTARRIHTELARMDN-QYMR  344
CiHQT2      VATDGRSRLSPRLPPGYLGNVVFTATPVAKSGDLTSGSLSNTAKLIHTTLTKMDD-DYLR  345
CiHQT3      VATDGRSRLSPQLPPGYLGNVVFTATPMAKSGELTTQPLSNSASLIRSTLSKMDN-EYLR  347
CiHCT1      IATDGRARLRPSLPPGYFGNVIFTTTPIAVAGELMSNPTWYAAGKIHDGLAKMDN-DYLK  340
CiHCT2      IATDGRARLQPALPPGYFGNVIFTTTPIAAAGDLQSKPTWYAASKIHDALVKMDN-DYLK  336
HaHQT1      VATDGRSRLNPPLPRGYLGNVIFTATPIMKVGEFKSESLGDTARRIHNELGRMNXXXYLR  341
HaHQT2      VATDGRSRLSPHLPRGYLGNVVFTATPVAKSGTLTSDSLVNAAKLIHTTLKKMDD-DYLR  346
HaHQT3      VATDGRSRLSPQLPPGYLGNVVFTATPIAKSADLTTQPLANAASLIRTTLSKMDN-DYLR  353
HaHCT1      IATDGRARLRPSLPPGYFGNVIFTTTPIAVAGDLQSKPTWYAAGKIHDALARMDN-DYLR  340
HaHCT2      IATDGRARLQPALPSGYFGNVIFTTTPVAVAGELQSKPTWYAASKIHEALAKMNN-DYLK  336
            :*****:** * ** **:***:**:**:     : :     :.  *:  * :*:   *::
```

Supplementary FIGURE S2 | Multiple sequence alignment of HQTs and HCTs from sunflower (HaHQTs and HaHCTs) and other representative species. Amino acid sequences of HaHQTs and HaHCTs are shown and aligned with those from other representative species including tomato (SlHQT; NP_001234850.2), globe artichoke (CcsHQT1; ABK79689.1, CcsHQT2; ADL62854.1, CcsHQT3; ADL62855.1, CcsHCT1; AAZ80046.1, and CcsHCT2; KVH99042.1), and chicory (CiHQT1; ANN12610.1, CiHQT2; ANN12611.1, CiHQT3; ANN12612.1, CiHCT1; ANN12608.1, and CiHCT2; ANN12609.1). (continue)

```
SlHQT       SALDYLELQPDLSTLIRGPAYFASPNLNINSWTRLPVHECDFGWGRPIHMGPACILYEGT  395
CcsHQT1     SAIDYLELQPDLTALVRGPTYFASPNLNINSWTRLPIYESDFGWGRPIFMGPASILYEGT  400
CcsHQT2     SAIDYLESQPDLSALIRGPSYFASPNLNINAWTRLPVYDADLGWGRPIFMGPACILYEGT  409
CcsHQT3     SAIDYPEVQPDLSALIRGPSYFASPNLNINTWTRLPVHDADFGWGRPVFMGPACILYEGT  398
CcsHCT1     SALDYLELQPDLKALVRGAHTFKCPNLGITSWARLPIHDADFGWGRPIFMGPGGIAYEGL  402
CcsHCT2     SALDYLELQPDLKALVRGAHTFKCPNLGITSWARLPIHDADFGWGRPIFMGPGGIAFEGL  396
CiHQT1      SAIDYLELQLDLSALVRGPTYFASPNLNVNSWTRLPLYESDFGWGRPIFMGPANILYEGT  404
CiHQT2      SAIDYLESQKDLSALIRGPSYFASPNLNINAWTRLPVHDADFGYGRPIFMGPAQILYEGT  405
CiHQT3      SAIDYLEVQPDLSALIRGPSYFASPNLNINTWTRLPVHDADFGWGRPVFMGPAVILYEGT  407
CiHCT1      SALDYLELQPDLKALVRGAHSFRCPNLGITSWARLPIHDADFGWGRPIFMGPGGIAYEGL  400
CiHCT2      SALDYLELQPDLKALVRGAHTFKCPNLGITSWARLPIHDADFGWGRPIFMGPGGIAFEGL  396
HaHQT1      SAIDYLESITDLSTLVRGPSYFASPNLNVNSWTRLPIYDSDFGWGRPIFMGPASILYEGT  401
HaHQT2      SAIDYLETQPDISALIRGPSYFASPNLNINAWTRLPVHDADFGWGRPIFMGPATILYEGT  406
HaHQT3      SAIDYLEVQPDLSALIRGPSYFASPNLNINTWTRLPVHDADFGWGRPVFMGPACILYEGT  413
HaHCT1      SALDYLELQPDLKALVRGAHTFKCPNLGITSWARLPIHDADFGWGRPIFMGPGGIAYEGL  400
HaHCT2      SALDYLELQSDLKALVRGAHTFKCPNLGITSWARLPIHDADFGWGRPIFMGPGGIAYEGL  396
            **:** *   *:.:*:**   * .***.:.:*:***::.*:*:***:.***. * :**
SlHQT       IYIIPSPNSKDRNLRLAVCLDAGHMSLFEKYLYEL  430
CcsHQT1     IYIIPSPSG-DRSVSLAVCLDPDHMSLFRKCLYDF  434
CcsHQT2     IYVLPSPNN-DRSVSLAVCLDANEQPLFEKFLYEF  443
CcsHQT3     IYVLPSPNN-DRSMSLAVCLDADEQPLFEKFLYDF  432
CcsHCT1     SFVLPSPIN-DGSLSIVISLQAEHMKLFSKFLYDI  436
CcsHCT2     SFVLPSPIN-DGSLSIAISLQSEHMKLFSKFLYDI  430
CiHQT1      IYIIPSPTD-DRSLKLAVCLDSDHMSLFQKYLYDF  438
CiHQT2      VYVLPSPNN-DRSVSLAVCLDANEQPLFEKFLYEM  439
CiHQT3      IYVLPSPNN-DRSMSLAVCLDADEQPLFEKFLYEF  441
CiHCT1      SFVLPSPIN-DGSLSIAISLQAEHMKVFSKFLYDI  434
CiHCT2      SFVLPSPVN-DGSLWIAISLLGEHMKLFSKFLYDI  430
HaHQT1      IYILPGPGG-DRSVALAVCLDPDHMALFKERLYDF  435
HaHQT2      VYVLPSPNN-DRSVSLAVCLDAKEQPLFEKYLYEL  440
HaHQT3      IYVLPSPNN-DRSMSLAVCLDADEQPLFEKFLYDF  447
HaHCT1      SFVLPSPDN-DGSLSIAISLQAEHMKLFSNLLYDI  434
HaHCT2      SFVLPSPIN-DGSLSIAISLQAQHMKLFSNYLYDI  430
             :::*.* . * .: :.:.*   .  :* : **::
```

Supplementary FIGURE S2 | Multiple sequence alignment of HQTs and HCTs from sunflower (HaHQTs and HaHCTs) and other representative species. Amino acid sequences of HaHQTs and HaHCTs are shown and aligned with those from other representative species including tomato (SlHQT; NP_001234850.2), globe artichoke (CcsHQT1; ABK79689.1, CcsHQT2; ADL62854.1, CcsHQT3; ADL62855.1, CcsHCT1; AAZ80046.1, and CcsHCT2; KVH99042.1), and chicory (CiHQT1; ANN12610.1, CiHQT2; ANN12611.1, CiHQT3; ANN12612.1, CiHCT1; ANN12608.1, and CiHCT2; ANN12609.1). (continue)

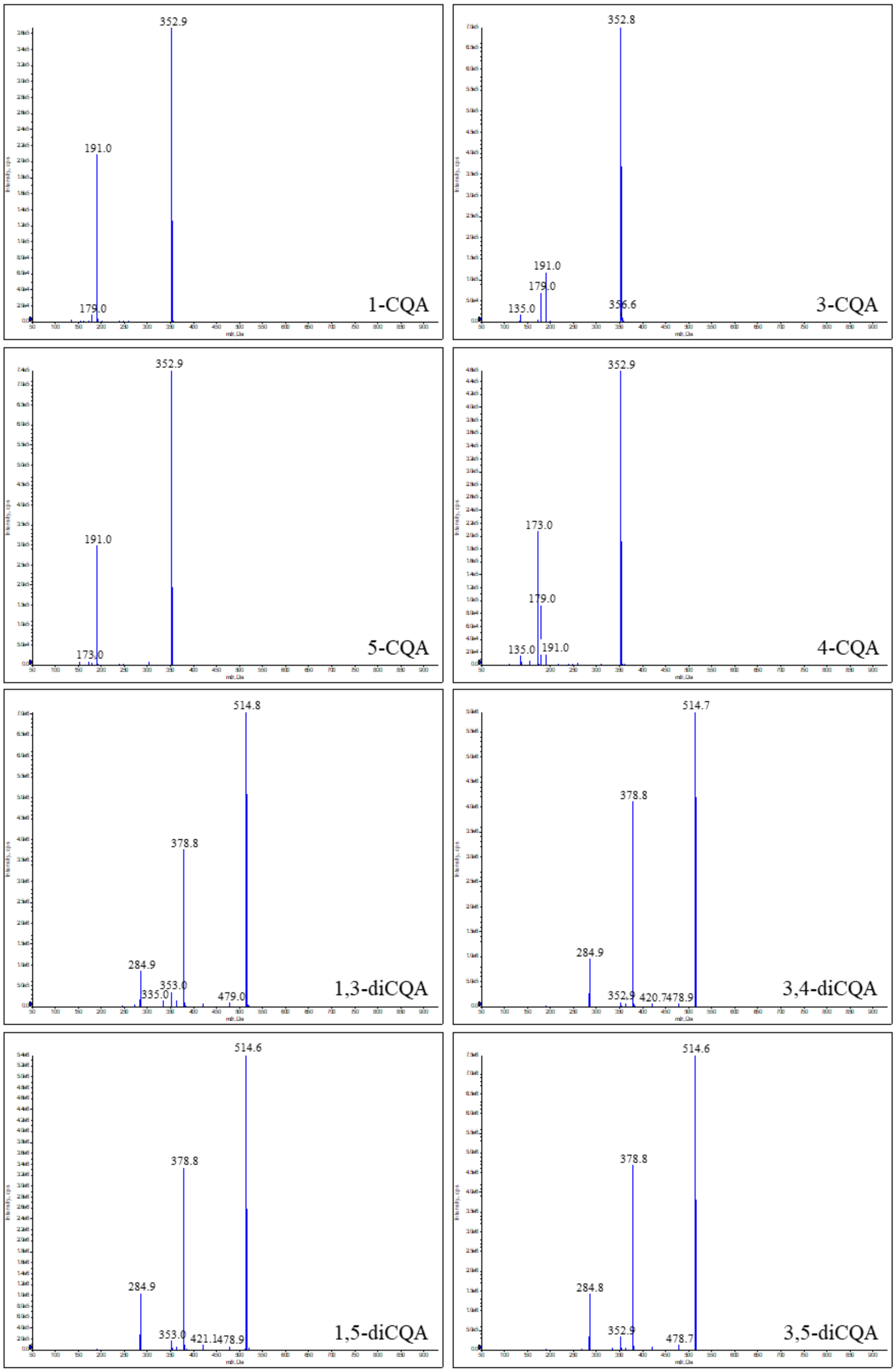

Supplementary FIGURE S3 | Negative ion MS^2 spectra of monocaffeoylquinic acids and dicaffeoylquinic acids. Enhanced Product Ion (EPI) profile of caffeoylquinic acid derivatives from authentic standards, including monnocaffeoylquinic acids such as 1-, 3-, 4- and 5-CQA, and diaffeoylquinic acids such as 1,3-, 1,5-, 3,4-, 3,5- and 4,5-diCQA.

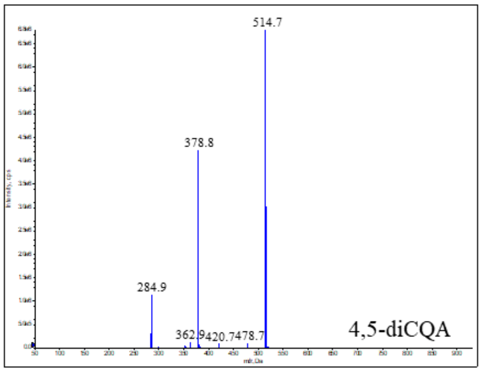

Supplementary FIGURE S3 | Negative ion MS^2 spectra of monocaffeoylquinic acids and dicaffeoylquinic acids. Enhanced Product Ion (EPI) profile of caffeoylquinic acid derivatives from authentic standards, including monnocaffeoylquinic acids such as 1-, 3-, 4- and 5-CQA, and diaffeoylquinic acids such as 1,3-, 1,5-, 3,4-, 3,5- and 4,5-diCQA. (continued)

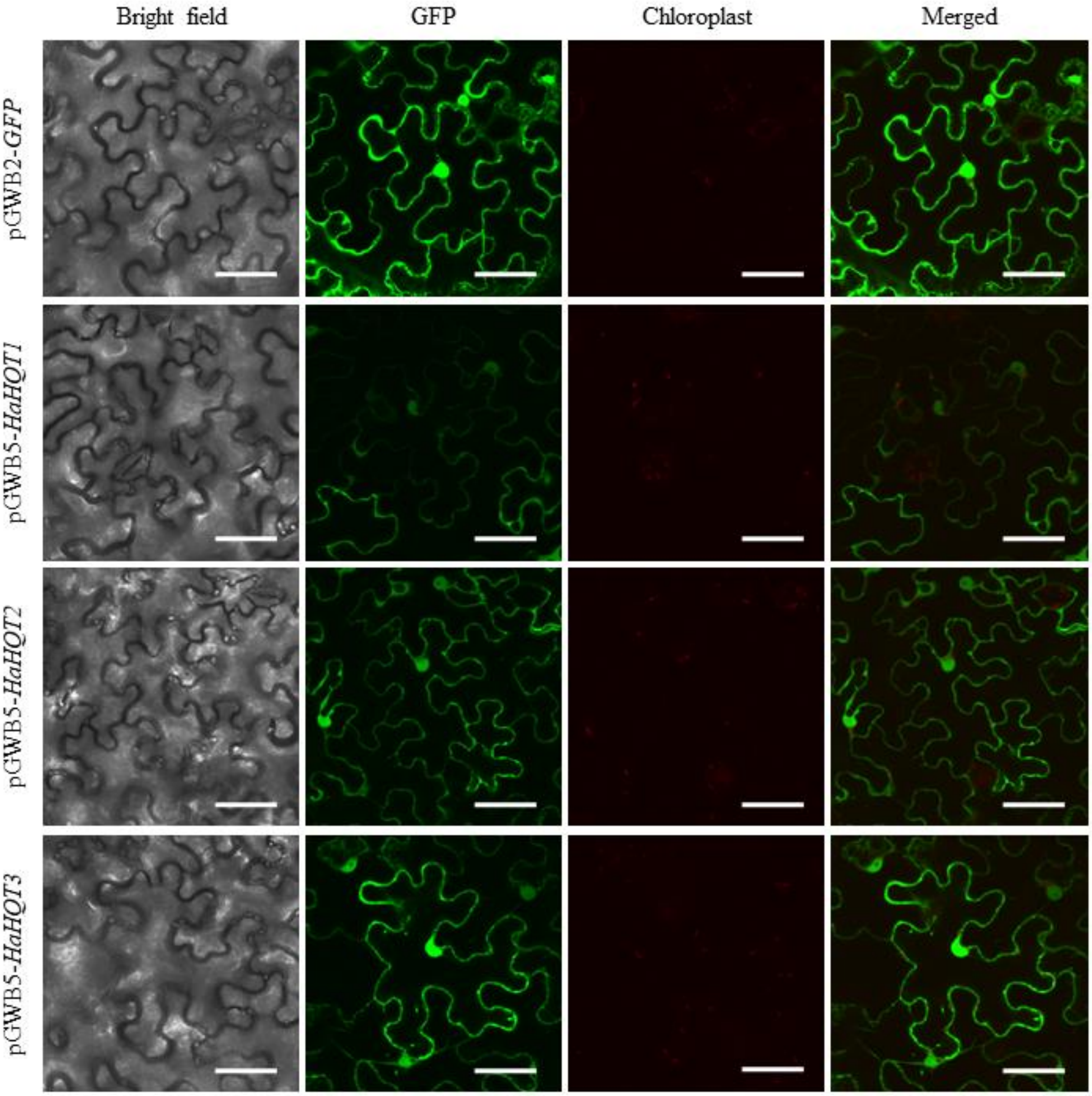

Supplementary FIGURE S4 | Subcellular localization of GFP-tagged HaHQTs in the epidermis of *Nicotiana benthamiana* leaves. Confocal microscopy image of epidermal cells from *N. benthamiana* leaves infiltrated with pGWB2-*GFP* (control), pGWB5-*HaHQT1*, pGWB5-*HaHQT2*, or pGWB5-*HaHQT3*. Bars=50 µm

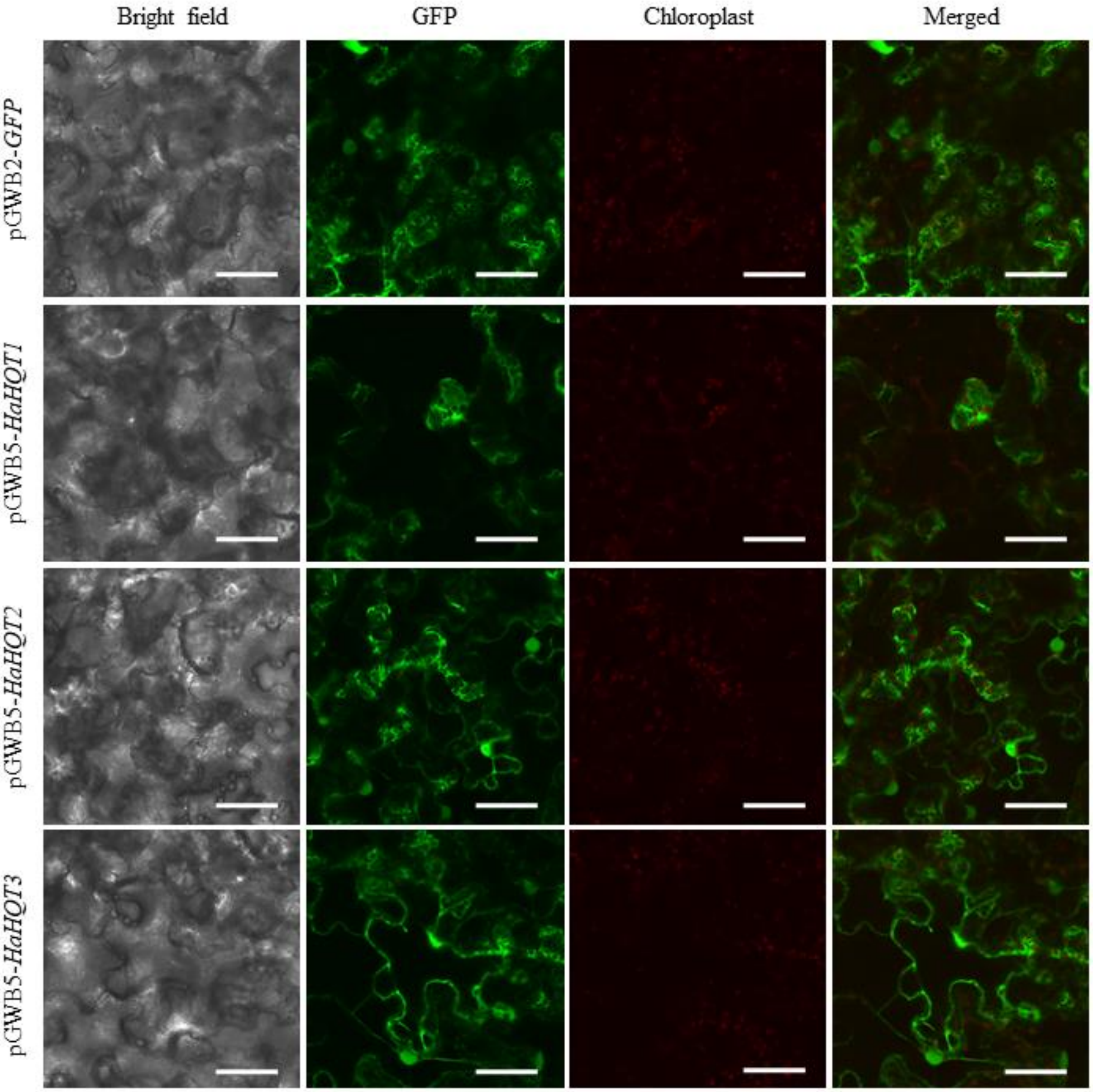

Supplementary FIGURE S5 | Subcellular localization of GFP-tagged HaHQTs in the mesophyll of *Nicotiana benthamiana* leaves. Confocal microscopy image of epidermal cells from *N. benthamiana* leaves infiltrated with pGWB2-*GFP* (control), pGWB5-*HaHQT1*, pGWB5-*HaHQT2*, or pGWB5-*HaHQT3*. Bars=50 μm

Supplementary Table S1 | All primers used in this study.

Experiment	Gene	Accession number	Primer Sequence (5'→3')		Product size (bp)
			Forward	Reverse	
Cloning	*HaHQT1*	MK598073	ATGAACCTAACAGTAAAACAATCACTAA	CTAGAAATCATACAAACGTTCCTTAAA	1,305
	HaHQT2	MK598074	ATGAAAACCGATCAAACCAAA	TTACAATTCATACAAGTACTTCTCAAAAA	1,323
	HaHQT3	MK598075	ATGGGGACTGTTCAAAAGACA	TTAGAAGTCGTATAAGAACTTCTCAAACA	1,326
Subcellular localization	*HaHQT1*	MK598073	ATGAACCTAACAGTAAAACAATCACTAA	GAAATCATACAAACGTTCCTTAAA	1,302
	HaHQT2	MK598074	ATGAAAACCGATCAAACCAAA	CAATTCATACAAGTACTTCTCAAAAA	1,320
	HaHQT3	MK598075	ATGGGGACTGTTCAAAAGACA	GAAGTCGTATAAGAACTTCTCAAACA	1,323
qRT-PCR /ddPCR	*ETIF5*	XM_022156448.1	TTGTGCCCTCGTCCCATAAC	AAATCTGCTCCTCCCCCATC	231
	EFI2	XM_022137686.1	TTGACCGCTAAGCCACGTTT	AGGCACCAGAAAACCCGAAT	199
	ACT7	XM_022154554.1	TTGGAATGGAAGCTGCTGGT	CTGGAGGAGCAACCACCTTG	201
	HaHQT1	MK598073	ACCGCAAGGAGAATCCACAA	CGTCCCCATCCAAAGTCAGA	194
	HaHQT2	MK598074	TGGTAAACGCTGCGAAACTG	GTCGCGGGTCCCATAAAAAT	223
	HaHQT3	MK598075	CCCCGATGACCAACTAACCA	GCTGATAGATCGGGCTGCAC	246
	HaHCT1	MK598076	GAGCTGCAGCCCGATCTAAA	CCGGGCTTGGTAACACAAAA	184
	HaHCT2	MK598077	CGGCGCTTGATTACTTGGAG	CACCAGGCCCCATGAATATG	159

Supplementary Table S2 | List of regulatory elements found in the promoter regions of *HaHQT2* and *HaHQT3*.

IUPAC family	Number of elements found		Function
	***HaHQT2* promoter**	***HaHQT3* promoter**	
P$FAM002	3	12	light responsiveness, auxin/ salicylic acid/ abscisic acid/ jasmonate responsiveness, stress regulation, dehydration responsiveness
P$FAM003	5	3	phenylpropanoid biosynthesis, phytochrome regulation, MYB
P$FAM008	1	1	water stress responsiveness, MYB
P$FAM010	8	4	elicitor responsiveness, defense response regulation, salicylic acid responsiveness, sucrose responsiveness
P$FAM012	3	7	light responsiveness, light regulation, MYB
P$FAM013	1	3	abscisic acid responsiveness, dehydration responsiveness, low temperature responsiveness
P$FAM014	4	8	phytochrome regulation, sugar repression, MYB
P$FAM015	0	2	sugar repression
P$FAM022	3	1	storage protein
P$FAM024	3	4	storage protein
P$FAM025	1	0	sugar repression
P$FAM026	1	0	light responsiveness
P$FAM027	2	1	light regulation, circadian rhythms
P$FAM061	1	1	pathogen, ethylene, and jasmonate responsiveness
P$FAM069	1	2	auxin responsiveness, sulfur responsiveness
P$FAM085	0	1	light responsiveness
P$FAM098	0	2	auxin responsiveness
P$FAM099	1	0	phytochrome responsiveness
P$FAM107	1	1	sugar starvation
P$FAM116	1	0	abscisic acid and drought responsiveness
P$FAM124	1	0	ethylene responsiveness
P$FAM162	2	1	low temperature responsive element
P$FAM170	1	1	gibberellin responsiveness, MYB
P$FAM171	1	5	MYB
P$FAM172	4	2	dehydration responsiveness
P$FAM205	0	2	gibberellin responsiveness and sugar repression, Dof
P$FAM227	2	0	storage protein
P$FAM234	0	1	storage protein
P$FAM243	2	6	storage protein
P$FAM244	2	0	storage protein
P$FAM245	0	2	storage protein
P$FAM251	1	0	light responsiveness
P$FAM261	2	2	low temperature responsiveness

Supplementary Table S2 | List of regulatory elements found in the promoter regions of *HaHQT2* and *HaHQT3*. (continue)

IUPAC family	Number of elements found		Function
	***HaHQT2* promoter**	***HaHQT3* promoter**	
P$FAM262	1	0	light responsiveness and circadian expression
P$FAM263	5	3	abscisic acid responsiveness
P$FAM266	6	2	dehydration responsiveness, MYB
P$FAM267	10	17	auxin responsiveness, Dof
P$FAM273	1	1	gibberellin responsiveness, sugar starvation
P$FAM290	7	4	pathogen- and salt-induced
P$FAM302	2	3	light responsiveness, axillary bud outgrowth
P$FAM310	2	1	cytokinin
P$FAM311	1	1	low CO_2, MYB
P$FAM315	0	1	light responsiveness
P$FAM324	0	2	calmodulin
P$FAM325	3	0	cell cycle, MYB
P$FAM329	0	1	wood formation
